# The important role of cuproptosis and cuproptosis-related genes in the development of thyroid carcinoma revealed by transcriptomic analysis and experiments

**DOI:** 10.1016/j.bjorl.2025.101560

**Published:** 2025-02-05

**Authors:** Yani Liu, Yanyan He, Shizhen Lei

**Affiliations:** a Wuhan No. 1 Hospital, Department of Otolaryngology and Head and Neck Surgery, Wuhan, China; b Shengjing Hospital of China Medical University, Department of Otolaryngology and Head and Neck Surgery, Shenyang, China; c Wuhan No. 1 Hospital, Department of Ophthalmology, Wuhan, China

**Keywords:** Thyroid carcinoma, Programmed cell death, Cuproptosis, CDKN2A, Tumor microenvironment, Immunosuppression

## Abstract

**Objective:**

Programmed Cell Death (PCD) processes have been suggested to play a role in the development of cancers. The study aimed to investigate the enrichment of different types of PCD and identify important PCD-related genes in Thyroid Carcinoma (THCA).

**Methods:**

The whole study is based on transcriptomic analysis and Immunohistochemistry (IHC) experiments. For transcriptomic analysis, the transcriptomic data of THCA (n = 493) and normal thyroid samples (n = 58) was used. The enrichment score of different types of PCD in THCA samples was calculated and the most enriched PCD process was identified. Then the expression of PCD-related genes in control and THCA samples were compared and the association between cuproptosis and characteristics of Tumor Microenvironment (TME) in THCA tissues was explored. IHC experiment was performed to confirm gene expressions in THCA and para-tumor samples.

**Results:**

Enrichment analysis showed that cuproptosis was the most enriched type of PCD process and associated with malignancy in THCA. The expression of a cuproptosis-related gene, CDKN2A, was found and confirmed to be higher in THCA than normal samples and associated with poor outcomes and higher clinical stages of patients with THCA. Moreover, CDKN2A expression was associated with immunosuppressive TME in THCA.

**Conclusion:**

Our findings indicated the important role of cuproptosis and a cuproptosis-related gene, CDKN2A, in the development and progression of THCA, which might provide novel insights into the understanding of pathophysiology of THCA.

## Highlights

• Cuproptosis was the most enriched type of Programmed Cell Death (PCD) processes in THCA.
• Cuproptosis was associated with tumor malignancy of THCA.
• A cuproptosis-related gene, CDKN2A, was upregulated in THCA sample.
• CDKN2A was associated with poor prognosis and TME in THCA.

## Introduction

Thyroid Carcinoma (THCA), derived from follicular epithelial cells or parafollicular epithelial cells of thyroid, has an increasing worldwide incidence and prevalence.^1^, ^2^ There were 586,202 newly diagnosed THCA cases in 2020, ranking 11th among all malignancies in terms of severity and accounting for 3.0% of all cancer patients.^3^ Although the death rate of THCA is relatively low, a small proportion of patients still suffer from an aggressive type with tumor progression and recurrence.^2^

The formation and maintenance of tissues, as well as the general health of multicellular organisms, depend on the essential and highly regulated process of cell death. It orchestrates both cell proliferation and elimination, ensuring a physiological balance in adult organisms. This intricate mechanism operates during critical stages such as metamorphosis, embryogenesis, and tissue turnover and in response to pathogenic threats.^4^ Programmed Cell Death (PCD) processes are highly regulated mechanisms of cellular self-destruction, which are the response to stimuli and controlled by multiple proteins and signaling cascades.^5^ There are six common and well-documented PCD types, including apoptosis, autophagy, ferroptosis, necroptosis, pyroptosis, and cuproptosis.^5^ Apoptosis, governed by signals, involves self-destruction in response to environmental or internal cues, playing a pivotal role in maintaining tissue homeostasis by eliminating unnecessary or damaged cells.^6^ Autophagy is a core molecular pathway for the preservation of cellular and organismal homeostasis. However, under stress and pathological conditions, excessive and uncontrolled autophagy can lead to over destruction of cell structure and finally cause autophagy-PCD.^7^ Ferroptosis refers to the non-apoptotic death of cells that accumulate lethal levels of iron-dependent, phospholipid peroxides in cell membranes.^8^ The necroptosis pathway is largely triggered by extracellular ligands that engage death receptors such as FAS Ligand (FASL) and FAS on the cell surface, which is a lytic form of cell death.^9^ Pyroptosis refers to cell death that is induced by gasdermin pores in the plasma membrane.^10^ Among them, cuproptosis is a newly discovered type of PCD, which is induced by disruptions in copper homeostasis^11^ and has been associated with the development of cancers.^12^ Dysregulation of these PCD processes has been correlated with tumors malignant features such as metastasis, recurrence, and mortality of patients.^13^, ^14^ However, our knowledge about PCD status and their clinical significance in THCA is still limited. Hence, exploring the effects of PCD is with great significance for understanding the mechanisms underlying the development of THCA.

In this study, we investigated the enrichment status of PCD processes in THCA and thus to identify important PCD process and PCD-related genes in THCA progression. As a result, cuproptosis was identified as the most enriched PCD process in THCA and a cuproptosis-related gene, CDKN2A, was associated with worse prognosis of patients with THCA. Immunohistochemistry (IHC) experiment confirmed the higher expression of CDKN2A in THCA samples than para-tumor samples. Collectively, our findings indicated the important role of cuproptosis and a cuproptosis-related gene, CDKN2A, in the development and progression of THCA.

## Methods

### Data acquisition of THCA samples

The transcriptomic data of THCA samples was obtained from the Cancer Genome Atlas (TCGA) database, which contains THCA (n = 493) and normal thyroid samples (n = 58). The gene expression data of samples was normalized by using the limma^15^ R package. Furthermore, we used the sva and limma R packages to remove the potential batch effect. The clinical information of these THCA samples was also obtained and used in further analysis.

### Enrichment analysis for PCD processes in THCA

Single-sample Gene Set Enrichment Analysis (ssGSEA)^16^ was performed to calculate the enrichment scores of the PCD and other well-known tumor progression-associated processes in each THCA samples. The PCD-related gene sets were obtained from Gene Ontology (GO)^17^ and Kyoto Encyclopedia of Genes and Genomes (KEGG)^18^ databases (Supplementary Table 1). Tumor progression-associated gene sets were also obtained from GO and KEGG databases, including Cell cycle, DNA damage, Epithelial-Mesenchymal Transition (EMT), Invasion, Stemness, and T-cell exhaustion (Supplementary Table 2).

### Cuproptosis and clinical characteristics in THCA

The clinical characteristics of THCA samples were visualized via ‘pheatmap’ R package, including tumor focus type, tumor width, tumor length, tumor depth, clinical stage, age at diagnosis, and gender.

### Expression of important cuproptosis-related genes across cancers

TIMER (Tumor IMmune Estimation Resource) database^19^ was used to obtain the expression of genes in cancers and GEPIA (Gene Expression Profiling Interactive Analysis) database was used to explore the association between gene expression and prognosis of patients with various cancers.

### Immunohistochemistry (IHC) experiment

Formalin-fixed, paraffin-embedded THCA and para-tumor tissues were collected from 40 patients with THCA diagnosed at our institution, from February 2023 to June 2024. Written informed consent was attained from patients before surgery. Three-micrometer-thick thyroid sections were prepared and deparaffinized, and then went through gradient alcohol hydration. According to the results of transcriptomic analysis and relative previous publications about cuproptosis and tumors,^20^, ^21^ we further validated the expression of a cuproptosis-related gene, CDKN2A, in THCA and para-tumor tissues by IHC experiments. The IHC experiment was conducted as the directions of the UltraSensitive IHC kit (cat. nº KIT-9710; Maixin, Fujian, China). The used primary antibody was polyclonal rabbit anti-CDKN2A (cat. nº 10883-1-AP; 1:500 dilution; proteintech, China). IHC results for CDKN2A expression were judged by the immunoreactive score, which was combined with the staining extent and intensity.

### Relevance of CDKN2A expression with TME characteristics in UM

TISIDB database provided the correlation of gene expression with TME characteristics in cancers, which was used to explore the relevance of CDKN2A expression with the expression of immune inhibitors and the infiltration of immune cells in THCA.

### Statistical analysis

The association between gene expression and patients’ prognosis was evaluated by univariate Cox regression analysis. Students’ *t*-test was used to examine the differences between groups.^22^ Spearman analysis was applied for correlation analysis. Analyses were mainly performed using the R and R Bioconductor packages.

## Results

### Cuproptosis was the most enriched PCD in THCA

**Fig. 1:**
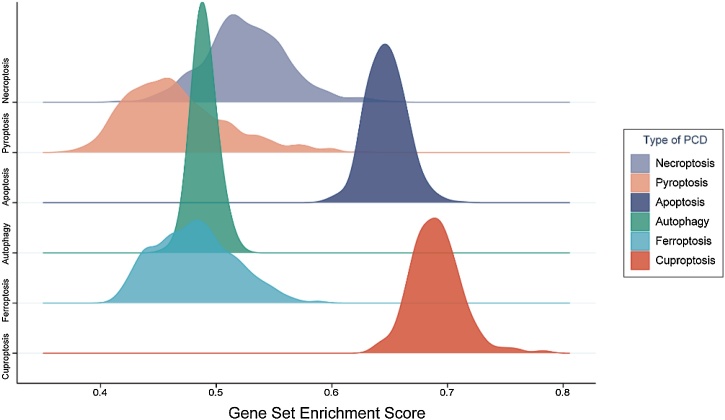
The enrichment scores of PCD processes in THCA samples calculated by ssGSEA analysis. PCD, Programmed Cell Death; THCA, Thyroid Carcinoma; ssGSEA, single-sample Gene Set Enrichment Analysis.

The ssGSEA analysis showed that cuproptosis had the highest enrichment score than other PCD types (Fig. 1, Supplementary Table 3), indicating that cuproptosis was the most enriched type of PCD process in THCA. Therefore, this study subsequently focused on cuproptosis and cuproptosis-related genes in THCA.

### Cuproptosis was associated with higher clinical stage and tumor progression in THCA

**Fig. 2:**
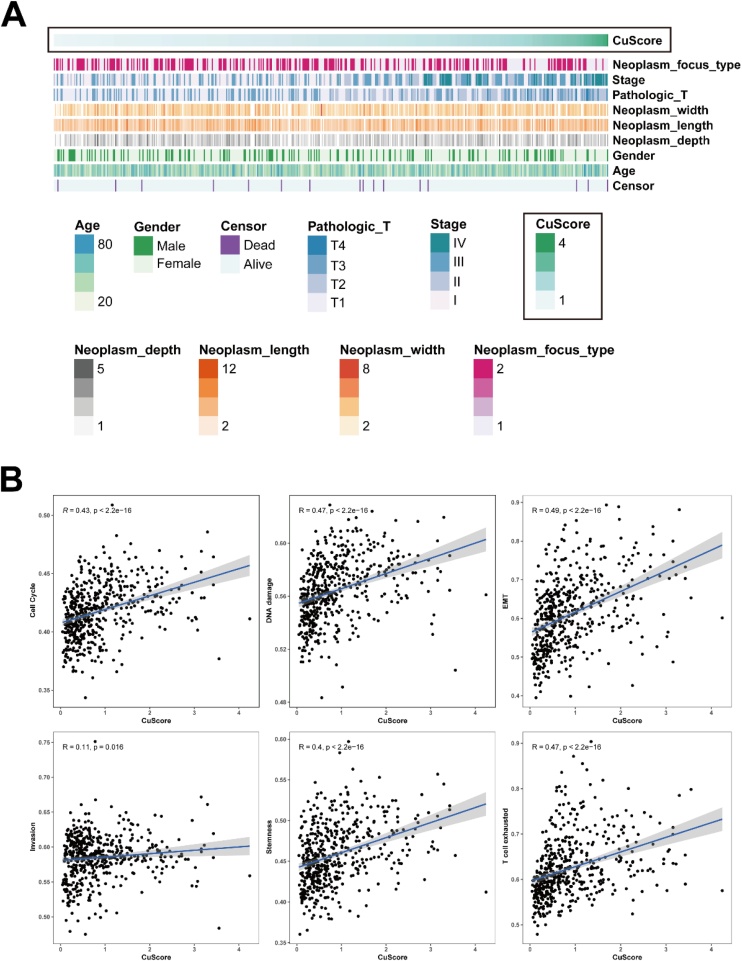
CuScore and tumor progression of THCA. (A) CuScore and clinical characteristics, including tumor focus type, tumor width, tumor length, tumor depth, clinical stage, age at diagnosis, and gender; (B) Correlation between CuScore and tumor progression-associated gene sets, including Cell cycle, DNA damage, EMT, Invasion, Stemness, and T-cell exhaustion. CuScore, Cuproptosis enrichment Score; THCA, Thyroid Carcinoma; EMT, Epithelial-Mesenchymal Transition.

The visualization of clinical information and cuproptosis enrichment score (CuScore) calculated by ssGSEA algorithm in 493 THCA samples indicated that CuScore was associated with higher stages of THCA (Fig. 2A). Additionally, correlation analysis showed that CuScore was significantly correlated with well-known tumor progression-associated processes (Fig. 2B).

### Identification of important cuproptosis-related genes in THCA

**Fig. 3:**
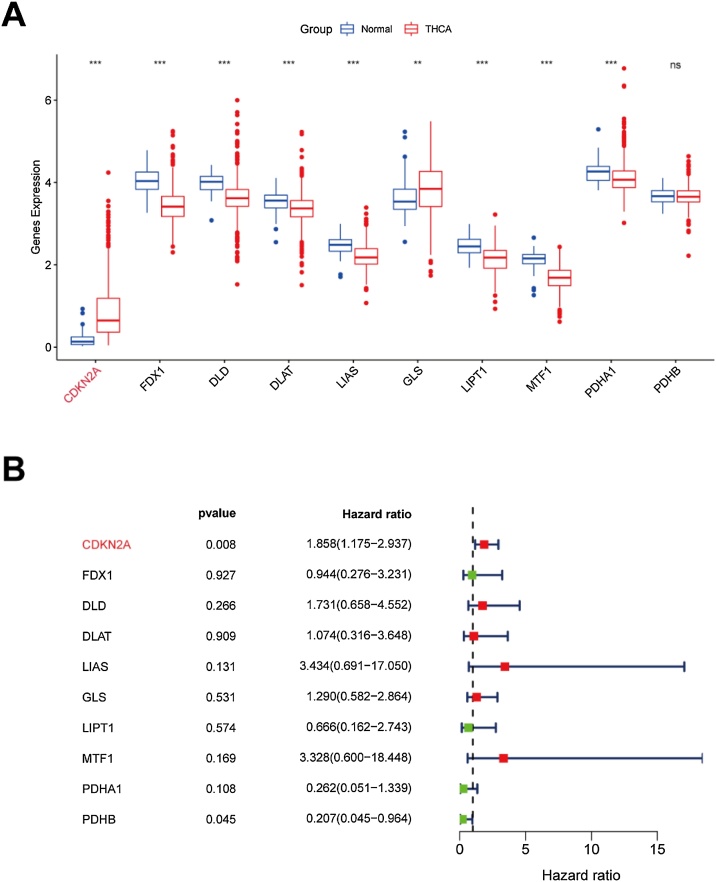
Expression of CRGs in THCA samples. (A) The comparison of CRGs expression in THCA and normal thyroid samples; (B) The prognostic value of CRGs evaluated by univariate Cox regression analysis. CRGs, Cuproptosis-Related Genes; THCA, Thyroid Carcinoma.

**Fig. 4:**
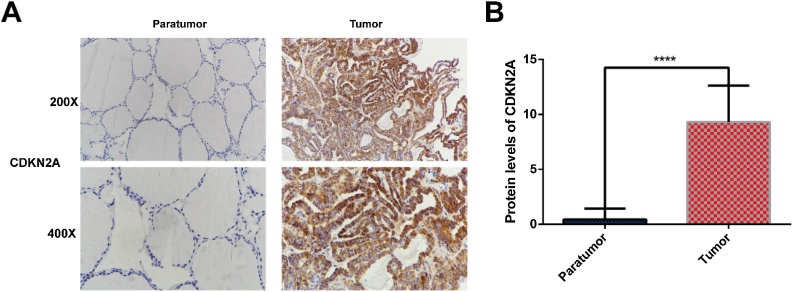
CDKN2A expression at protein level in THCA and para-tumor tissues by IHC experiment. THCA, Thyroid Carcinoma; IHC, Immunohistochemistry.

The expression of a cuproptosis-related gene, CDKN2A, was significantly higher in THCA than normal samples (Fig. 3A) and associated with poor outcomes of patients with THCA (Fig. 3B). Moreover, results of IHC experiment also supported the higher expression of CDKN2A in THCA samples (Fig. 4).

### CDKN2A expression in cancers

**Fig. 5:**
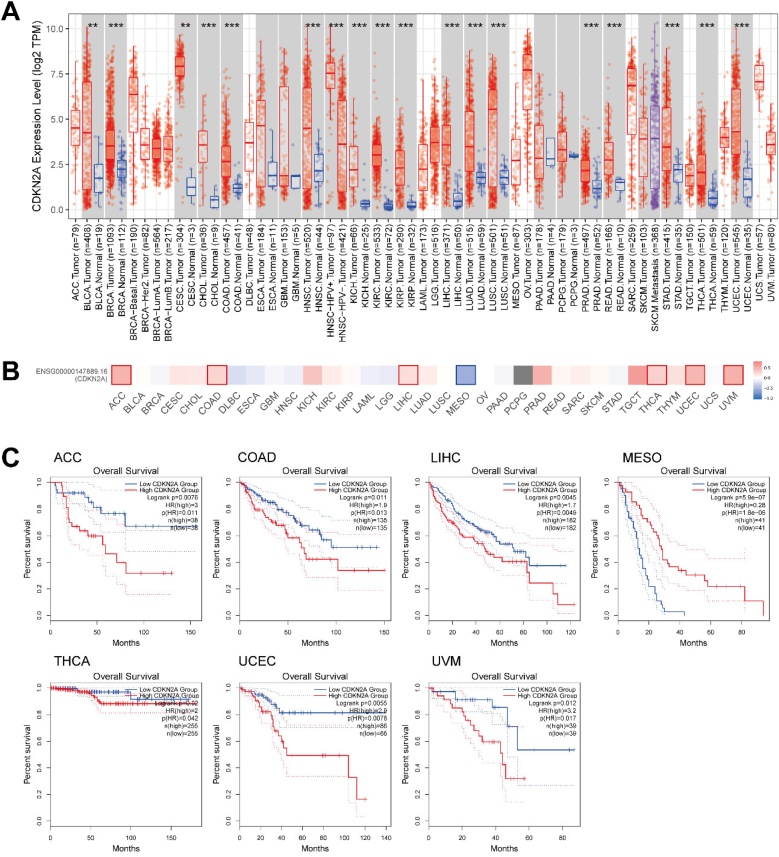
CDKN2A expression in cancers. (A) The box plot showing the expression of CDKN2A in cancers from TIMER 2.0 database; (B‒C) The prognostic analysis results for CDKN2A in cancers from GEPIA 2.0 database.

Expression profile of CDKN2A in cancers from TIMER database supported the higher expression of CDKN2A in THCA samples than normal thyroid tissues (Fig. 5A) and the association between CDKN2A expression and worse prognosis of patients with THCA (Fig. 5B‒C). Interestingly, CDKN2A expression was correlated with worse prognosis of patients with other cancers, including Adrenocortical Carcinoma (ACC), Colon Adenocarcinoma (COAD), Liver Hepatocellular Carcinoma (LIHC), Uterine Corpus Endometrial Carcinoma (UCEC), and Uveal melanoma (UVM) (Fig. 5C).

### CDKN2A expression was tightly associated with immunosuppressive TME in THCA

**Fig. 6:**
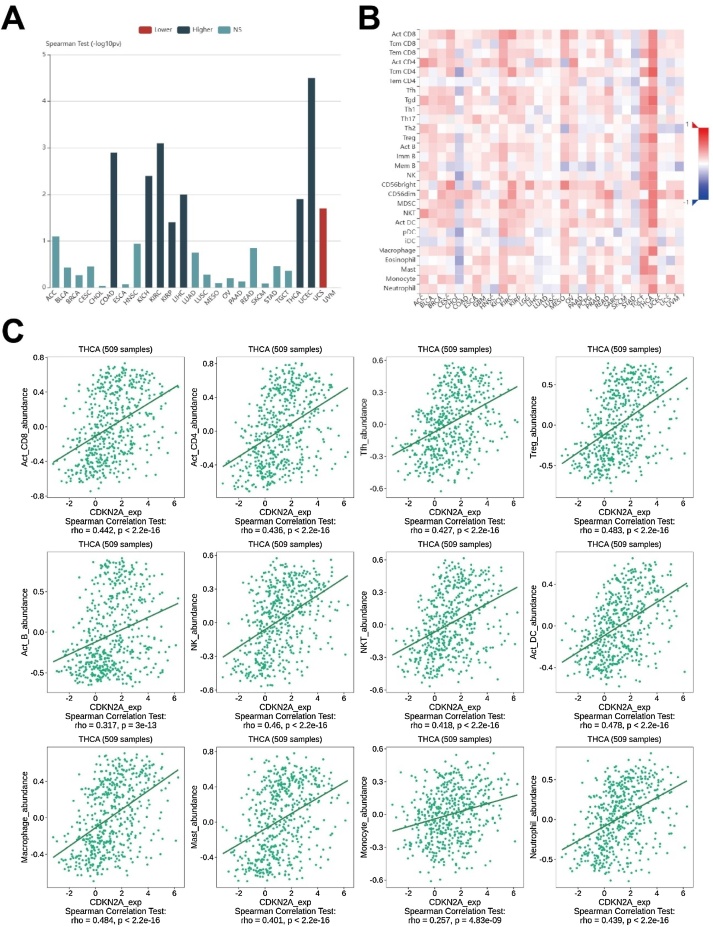
Association between CDKN2A expression and tumor stage and immune cell infiltration in THCA from TISIDB database. (A) CDKN2A expression was associated with higher stages in THCA; (B‒C) CDKN2A expression and the abundance of tumor-infiltrating immune cells in THCA. THCA, Thyroid Carcinoma.

**Fig. 7:**
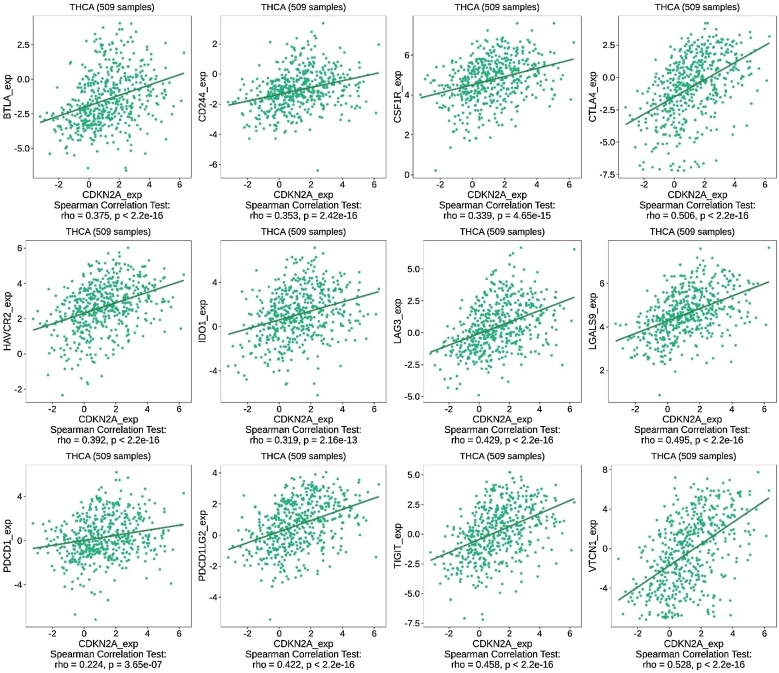
Association between the expression of CDKN2A and immune inhibitors, including VTCN1, TIGIT, PD1LG2, PD1, LGALS9, LAG3, IDO1, HAVCR2, CTLA4, CSF1R, CD244, and BTLA, obtained from TISIDB database.

Firstly, results from TISIDB supported the correlation between CDKN2A expression and higher stage of THCA (Fig. 6A). Furthermore, results from TISIDB indicated that CDKN2A expression was positively correlated with the abundance of tumor-infiltrating immune cells including Activated CD8 T-cell (Act CD8), Activated CD4 T-cell (Act CD4), T-follicular helper cell (Tfh), Regulatory T-cell (Treg), Activated B cell (Act B), Natural Killer cell (NK), Natural Killer T-cell (NKT), Activated Dendritic Cell (Act DC), Macrophage, Mast cell, Monocyte, and Neutrophil in THCA (Fig. 6B‒C). Importantly, CDKN2A expression was significantly positively associated with the expression of immune inhibitors, including VTCN1, TIGIT, PD1LG2, PD1, LGALS9, LAG3, IDO1, HAVCR2, CTLA4, CSF1R, CD244, and BTLA (Fig. 7).

## Discussion

Dysregulation of PCD processes has been implicated in the malignant features and progression of tumors.^13^, ^14^ Notably, the important role of cuproptosis in cancer development has been identified.^12^ However, the role of cuproptosis and cuproptosis-related genes in THCA microenvironment and development is still unclear. Here in this study, we found that cuproptosis was the most enrich PCD process in THCA and identified the potential role of cuproptosis in the progression of THCA.

Copper is a microelement essential for organisms including human beings and vital in various biological processes.^23^ Copper homeostasis disruption can cause cellular toxicity and cell death, which is named as cuproptosis^24^, ^25^, ^26^ and has been implicated in the development and progression of cancers.^12^ Excessive copper can influence tricarboxylic acid cycle, leading to stress-inducing proteins accumulation and causing cell death.^27^ Considering the function of copper as an inducer of cellular toxicity, copper accumulation can be the target for eliminating tumor cells and show potential as a therapeutic target for cancers. In fact, based on this mechanism, various agents with the ability to influence copper metabolism such as Disulfiram and Sanchoishi have been used in cancer treatment.^28^, ^29^, ^30^ Notably, copper can also promote angiogenesis and subsequent tumor growth, by stimulating pro-angiogenic signals.^31^, ^32^ In addition, copper can activate the BRAF signaling pathway in cancers, thereby promoting tumor cell proliferation and migration.^32^ Notably, here in this study, Cuproptosis enrichment Score (CuScore) was found to be positively correlated with well-known tumor progression-associated processes, including Cell cycle, DNA damage, EMT, Invasion, Stemness, and T-cell exhaustion. Given the dual role of copper in cancer development reported by previous studies and the findings in this study, further exploring the effects and underlying mechanisms of cuproptosis in THCA is of great significance in understanding the mechanisms underlying its progression.

We evaluated the prognostic value of cuproptosis-related genes and found that CDKN2A was significantly associated with worse prognosis of patients with THCA. Higher CDKN2A expression was identified and confirmed by IHC experiment in THCA than normal thyroid tissues. Moreover, CDKN2A expression was significantly associated with higher clinical stages of THCA. CDKN2A is an unstable gene, whose alterations are frequently observed in cancers, such as head and neck cancer, melanoma, and lung cancers.^33^ The epigenetic or genetic changes of CDKN2A has also been associated with the initiation of ovarian cancer and melanoma.^34^, ^35^ It has been reported that CDKN2A alteration is related to immunotherapy resistance in cancers.^21^, ^36^, ^37^ In addition, previous studies have speculated on an association between CDKN2A and copper homeostasis in cuproptosis.^38^, ^39^ Moreover, Cheng et al. found that CDKN2A mediates cuproptosis resistance through regulating glycolysis and copper homeostasis and is associated with the progression of colorectal cancer.^20^

Tumor Microenvironment (TME) is crucial to tumor progression and immunotherapy efficacy and resistance in cancers.^40^, ^41^ Importantly, CDKN2A was significantly correlated with TME characteristics in THCA. Although CDKN2A expression was positively associated with the infiltration of tumor-killing CD8 T-cells, it was also positively correlated with immunosuppressive cells such as regulatory T-cells (Treg) and immunoinhibiting factors such as PD1 and LAG3, indicating that higher CDKN2A expression has the potential to induce immunosuppressive TME and T-cell allergy, thereby leading to the malignant features and poor prognosis of patients with THCA.

## Conclusion

In conclusion, our findings suggested the important role of cuproptosis and a cuproptosis-related gene, CDKN2A, in the development and progression of THCA, which might be bridged by their influence on TME characteristics. However, the exact mechanisms underlying their effects in THCA are still required to be elucidated by further studies.

## Ethics

This study was approved by the Ethics Committee of Wuhan No. 1 Hospital. Our research adhered to the tenets of the Declaration of Helsinki.

## Funding

This study was supported by The Funding for Scientific Research Projects from Wuhan Municipal Health Commission (WX23Z33).

## Declaration of competing interest

The authors declare no conflicts of interest.
