# Supplementary material for: The important role of cuproptosis and cuproptosis-related genes in the development of thyroid carcinoma revealed by transcriptomic analysis and experiments
Source: Braz J Otorhinolaryngol. 2025 Feb 5;91(3):101560. doi: 10.1016/j.bjorl.2025.101560 (PMC11848474; doi:10.1016/j.bjorl.2025.101560)
Supplement: Supplementary file 1 [file mmc1.docx]

**BJORL-D-24-00308_Supplementary Material**

**SUPPLEMENTAL TABLES**

**The important role of cuproptosis and cuproptosis-related genes in the development of thyroid carcinoma revealed by transcriptomic analysis and experiments**

Contents

[Supplemental Table 1. Gene sets of Programmed cell death processes 1](#_Toc184750180)

[Supplemental Table 2. Gene sets of well-known tumor progression-associated processes 4](#_Toc184750181)

[Supplemental Table 3. PCD scores of THCA samples. 10](#_Toc184750182)

Supplemental Table 1. Gene sets of Programmed cell death processes

|  | **Necroptosis** | **Pyroptosis** | **Apoptosis** | **Autophagy** | **Ferroptosis** | **Cuproptosis** |
| --- | --- | --- | --- | --- | --- | --- |
| **Gene** | RIPK3  MLKL  FAS  FASLG  TLR3  TNF  RIPK1  FADD | AIM2  APIP  CASP1  CASP4  CASP6  CASP8  DHX9  ELANE  GSDMA  GSDMB  GSDMC  GSDMD  GSDME  GZMA  GZMB  NAIP  NLRC4  NLRP1  NLRP6  NLRP9  TREM2  ZBP1 | AIFM1  AKT1  AKT2  AKT3  APAF1  ATM  BAD  BAX  BCL2  BCL2L1  BID  BIRC2  BIRC3  CAPN1  CAPN2  CASP10  CASP3  CASP6  CASP7  CASP8  CASP9  CFLAR  CHP1  CHP2  CHUK  CSF2RB  CYCS  DFFA  DFFB  ENDOD1  ENDOG  EXOG  FADD  FAS  FASLG  IKBKB  IKBKG  IL1A  IL1B  IL1R1  IL1RAP  IL3  IL3RA  IRAK1  IRAK2  IRAK3  IRAK4  MAP3K14  MYD88  NFKB1  NFKBIA  NGF  NTRK1  PIK3CA  PIK3CB  PIK3CD  PIK3CG  PIK3R1  PIK3R2  PIK3R3  PIK3R5  PPP3CA  PPP3CB  PPP3CC  PPP3R1  PPP3R2  PRKACA  PRKACB  PRKACG  PRKAR1A  PRKAR1B  PRKAR2A  PRKAR2B  PRKX  RELA  RIPK1  TNF  TNFRSF10A  TNFRSF10B  TNFRSF10C  TNFRSF10D  TNFRSF1A  TNFSF10  TP53  TRADD  TRAF2  XIAP | ATG12  ATG3  ATG4A  ATG4B  ATG4C  ATG4D  ATG5  ATG7  BECN1  BECN2  GABARAP  GABARAPL1  GABARAPL2  IFNA1  IFNA10  IFNA13  IFNA14  IFNA16  IFNA17  IFNA2  IFNA21  IFNA4  IFNA5  IFNA6  IFNA7  IFNA8  IFNG  INS  PIK3C3  PIK3R4  PRKAA1  PRKAA2  ULK1  ULK2  ULK3 | ACSL1  ACSL3  ACSL4  ACSL5  ACSL6  ALOX15  ATG5  ATG7  CP  CYBB  FTH1  FTL  FTMT  GCLC  GCLM  GPX4  GSS  HMOX1  LPCAT3  MAP1LC3A  MAP1LC3B  MAP1LC3C  NCOA4  PCBP1  PCBP2  PRNP  SAT1  SAT2  SLC11A2  SLC39A14  SLC39A8  SLC3A2  SLC40A1  SLC7A11  STEAP3  TF  TFRC  TP53  VDAC2  VDAC3 | CDKN2A  FDX1  DLD  DLAT  LIAS  GLS  LIPT1  MTF1  PDHA1  PDHB |

Supplemental Table 2. Gene sets of well-known tumor progression-associated processes

|  | **Cell Cycle** | **DNA damage** | **EMT** | **Invasion** | **Stemness** | **T cell exhausted** |
| --- | --- | --- | --- | --- | --- | --- |
| **Gene** | ACVR1  ACVR1B  AKAP8  ANLN  APBB1  APBB2  AURKA  BCAT1  BIRC5  BOLL  BRSK1  CD28  CDCA5  CDK10  CDK13  CDK2AP1  CDKN3  CENPE  CENPF  CETN1  CHFR  CHMP1A  CIT  CLIP1  CUL2  CUL3  CUL4A  CUL5  DCTN2  DCTN3  DDX11  DLGAP5  DMC1  DUSP13  EGF  EPGN  EREG  FOXN3  FOXO4  GFI1  GFI1B  GML  GSPT1  HSPA2  INHBA  KATNA1  KHDRBS1  KIF11  KIF15  KIF22  KIF25  KIF2C  KNTC1  KPNA2  KRT7  LATS1  LATS2  LIG3  MAP3K11  MPHOSPH6  MPHOSPH9  MRE11  MSH4  MSH5  MYO16  NBN  NCAPH  NDC80  NEK2  NEK6  NOLC1  NPM2  NUMA1  NUSAP1  P3H4  PAM  PBRM1  PCBP4  PDS5B  PIM2  PIN1  PML  POLA1  POLD1  POLE  PPP5C  PPP6C  PRMT5  PRUNE2  PTPRC  RAD1  RAD17  RAD50  RAD51  RAD51B  RAD51D  RAD52  RAD54B  RAD54L  RAN  RCC1  REC8  RINT1  SMC4  SPDYA  SPO11  STAG3  SUGT1  SYCP1  TAF1  TAF1L  TARDBP  TBRG4  TGFA  TIMELESS  TIPIN  TOP3A  TPD52L1  TPX2  TRIAP1  TTN  UBE2C  USH1C  XRCC2  ZNRD2  ZW10  ZWINT  AC023512.1  ANAPC1  ANAPC13  ANAPC2  ANAPC7  ATR  BUB3  CCNB1  CCNB2  CCNB3  CCND1  CCND2  CCND3  CCNE1  CCNE2  CCNH  CDC14A  CDC14B  CDC20  CDC25A  CDC26  CDC45  CDK1  CDK7  CHEK2  CREBBP  E2F2  E2F3  E2F4  E2F5  EP300  FZR1  GADD45A  GADD45B  GADD45G  GSK3B  HDAC1  HDAC2  MAD1L1  MCM2  MCM3  MCM4  MCM5  MCM6  MCM7  MDM2  MYC  ORC1  ORC2  ORC3  ORC4  ORC5  ORC6  PCNA  PRKDC  PTTG1  PTTG2  RBL1  RBL2  RBX1  SFN  SKP1  SMAD2  SMAD3  SMAD4  SMC1B  STAG1  STAG2  TFDP1  TFDP2  TGFB2  TGFB3  TP53  WEE1  WEE2  YWHAB  YWHAE  YWHAG  YWHAH  YWHAQ  YWHAZ  ZBTB17  ANAPC10  ANAPC11  ANAPC4  ANAPC5  ATM  BUB1  BUB1B  CCNA1  CCNA2  CDC16  CDC23  CDC25B  CDC25C  CDC27  CDC6  CDC7  CDK2  CDK4  CDK6  CDKN1A  CDKN1B  CDKN1C  CDKN2A  CDKN2B  CDKN2C  CDKN2D  CHEK1  CUL1  DBF4  E2F1  ESPL1  MAD2L1  MAD2L2  PKMYT1  PLK1  RAD21  RB1  SKP2  SMC1A  SMC3  TGFB1  TTK | ACER2  ANKRD1  ARID3A  ATM  ATR  ATRX  AURKA  BATF  BAX  BCL3  BRCA1  BRCA2  BTG2  CARM1  CASP2  CCNB1  CD44  CD74  CDC25C  CDK1  CDK2  CDKN1A  CDKN1B  CHEK2  CNOT1  CNOT10  CNOT11  CNOT2  CNOT3  CNOT4  CNOT6  CNOT6L  CNOT7  CNOT8  CNOT9  CRADD  DDX5  DYRK1A  DYRK3  E2F1  E2F4  E2F7  E2F8  EEF1E1  EP300  FOXM1  FOXO3  GADD45A  GML  GTSE1  HIC1  HIPK2  ING4  KAT5  KDM1A  KMT5A  MARCHF7  MDM2  MDM4  MIF  MSX1  MUC1  MYO6  NBN  NDRG1  PAXIP1  PCBP4  PCNA  PIDD1  PLA2R1  PLAGL1  PLK2  PLK3  PMAIP1  PML  PPM1D  PRMT1  PSMD10  PTTG1IP  PYHIN1  RBL2  RGCC  RPL26  RPS27L  RPS6KA6  SESN2  SFN  SIRT1  SMYD2  SNAI1  SNAI2  SOX4  SP100  SPRED1  SPRED2  SPRED3  TFAP4  TFDP1  TFDP2  TNKS1BP1  TP53  TRIAP1  TWIST1  USP10  YJU2  ZMPSTE24  ZNF385A | ACTA2  ADAM12  ANPEP  APLP1  AREG  BASP1  BDNF  BGN  BMP1  CADM1  CALD1  CALU  CAP2  CAPG  CCN1  CCN2  CD44  CD59  CDH11  CDH2  CDH6  COL11A1  COL12A1  COL16A1  COL1A1  COL1A2  COL3A1  COL4A1  COL4A2  COL5A1  COL5A2  COL5A3  COL6A2  COL6A3  COL7A1  COL8A2  COLGALT1  COMP  COPA  CRLF1  CTHRC1  CXCL1  CXCL12  CXCL6  CXCL8  DAB2  DCN  DKK1  DPYSL3  DST  ECM1  ECM2  EDIL3  EFEMP2  ELN  EMP3  ENO2  FAP  FAS  FBLN1  FBLN2  FBLN5  FBN1  FBN2  FERMT2  FGF2  FLNA  FMOD  FN1  FOXC2  FSTL1  FSTL3  FUCA1  FZD8  GADD45A  GADD45B  GAS1  GEM  GJA1  GLIPR1  GPC1  GPX7  GREM1  HTRA1  ID2  IGFBP2  IGFBP3  IGFBP4  IL15  IL32  IL6  INHBA  ITGA2  ITGA5  ITGAV  ITGB1  ITGB3  ITGB5  JUN  LAMA1  LAMA2  LAMA3  LAMC1  LAMC2  LGALS1  LOX  LOXL1  LOXL2  LRP1  LRRC15  LUM  MAGEE1  MATN2  MATN3  MCM7  MEST  MFAP5  MGP  MMP1  MMP14  MMP2  MMP3  MSX1  MXRA5  MYL9  MYLK  NID2  NNMT  NOTCH2  NT5E  NTM  OXTR  P3H1  PCOLCE  PCOLCE2  PDGFRB  PDLIM4  PFN2  PLAUR  PLOD1  PLOD2  PLOD3  PMEPA1  PMP22  POSTN  PPIB  PRRX1  PRSS2  PTHLH  PTX3  PVR  QSOX1  RGS4  RHOB  SAT1  SCG2  SDC1  SDC4  SERPINE1  SERPINE2  SERPINH1  SFRP1  SFRP4  SGCB  SGCD  SGCG  SLC6A8  SLIT2  SLIT3  SNAI2  SNTB1  SPARC  SPOCK1  SPP1  TAGLN  TFPI2  TGFB1  TGFBI  TGFBR3  TGM2  THBS1  THBS2  THY1  TIMP1  TIMP3  TNC  TNFAIP3  TNFRSF11B  TNFRSF12A  TPM1  TPM2  TPM4  VCAM1  VCAN  VEGFA  VEGFC  VIM  WIPF1  WNT5A | AMPD3  CA2  CSF3R  MAGEA9  OGG1  RAB39A  RPAP1  RYR1  SERPINB6  TAF4B  TRIM8  UBE3C  ZNF687  CAPG  CD44  CTSL  DNMT1  EGFR  ENPP2  EZR  HDAC3  KLHL41  LGALS3  MMP1  SPARC  STAT6 | A1BG  A2M  AGFG1  ANKRD27  ANLN  APOBEC3B  ARF1  ASF1A  ASPM  ATAD2  AURKA  AVL9  BIRC5  BUB1  C1orf112  CCNB2  CDC123  CDCA5  CDCA7  CDK1  CDKN3  CDT1  CEBPG  CENPA  CENPE  CENPF  CENPM  CENPN  CENPU  CHAF1B  CKS1B  CKS2  CMC2  CMSS1  CNIH4  COX5A  CSE1L  CSNK1G1  CSTB  CTPS1  CTSV  CYRIB  DAP3  DBF4  DCUN1D5  DEK  DESI2  DTL  DTYMK  EMC8  EXO1  EZH2  FAM3C  FANCA  FANCE  GARS1  GART  GDI2  GGCT  GINS3  GNB4  GPSM2  GTPBP4  H2AZ1  H2AZ2  HDAC2  HIKESHI  HSPA14  IDH2  ILF2  KDELR2  KNSTRN  KPNA2  LBR  LGALS8  LRP8  MAD2L1  MAGOHB  MARCO  MCM2  MCM3  MND1  MRPL9  MSH2  MTFR2  MYBL2  NCAPG  NCS1  NDC80  NDUFB5  NEK2  NFE2L3  NMI  NUDT5  NUF2  PARP1  PBK  PCNA  PDCD10  PDCD5  PDSS1  PFDN2  PKMYT1  PLAAT1  PPIL1  PRC1  PRIM2  PROSER1  PRPF18  PSMA3  PSMB4  PTS  PTTG1  PURB  RACGAP1  RAD51  RAD51AP1  RAD54L  RANBP1  RBM8A  RDX  RFC4  RIT1  RRAGD  RRM2  S100A11  S100A9  SEM1  SLC25A5  SMAD2  SNRPD1  SNRPG  SPAG5  SRPK1  STMN1  SUV39H2  TFRC  TMEM14A  TMSB10  TOP2A  TP53BP2  TTK  TYMS  UBE2T  UGGT1  UTP25  WDR26 | HAVCR2  CXCL13  CCL3  SIRPG  IFNG  TIGIT  GZMB  PDCD1  PARK7  TNFRSF9  ACP5  CTLA4  RBPJ  MIR155  CXCR6  CD27  FKBP1A  BST2  TPI1  MIR155HG  PTTG1  CD63  SAMSN1  RGS1  CD27-AS1  ITGAE  MIR4632  HLA-DRA  IGFLR1  KRT86  ENTPD1  DUSP4  SIT1  TOX  PHLDA1  CCND2  GPR25  LAYN  PRDX5  SARDH  FASLG  MIR3917  ANXA5  CTSD  PDIA6  RANBP1  FKBP1A-SDCBP2  COTL1  TNFRSF1B  IDH2  CD38  CD82  LAG3  MIR497HG  APOBEC3C  ITM2A  COX5A  IFI35  NDFIP2  TNFRSF18  KRT81  DNPH1  RGS2  HMGN1  DYNLL1  SNRPB  STRA13  SYNGR2  RAB27A  PSMC3  GALM  FABP5  UBE2L6  MYO7A  PRDX3  DDIT4  STMN1  CDK2AP2  VCAM1  SNAP47  PSMB3  ISG15  HLA-DRB5  CKS2  TNIP3  CD7  PSMD4  ATP6V1C2  PSMD8  HLA-DRB6 |

Supplemental Table 3. PCD scores of THCA samples.

| id | Necroptosis | Pyroptosis | Apoptosis | Autophagy | Ferroptosis | Cuproptosis |
| --- | --- | --- | --- | --- | --- | --- |
| TCGA-4C-A93U-01A | 0.566418274 | 0.550475028 | 0.655276149 | 0.493082969 | 0.519798481 | 0.7089798 |
| TCGA-BJ-A0YZ-01A | 0.556661744 | 0.420835922 | 0.646922891 | 0.49136329 | 0.446236206 | 0.7124826 |
| TCGA-BJ-A0Z0-01A | 0.470121437 | 0.408540287 | 0.623249264 | 0.493472972 | 0.428568649 | 0.6775794 |
| TCGA-BJ-A0Z3-01A | 0.535338629 | 0.481478185 | 0.661992952 | 0.473157884 | 0.509268586 | 0.682809 |
| TCGA-BJ-A0Z5-01A | 0.505914245 | 0.447544524 | 0.636788581 | 0.483890701 | 0.466054294 | 0.6801573 |
| TCGA-BJ-A0Z9-01A | 0.551466573 | 0.471747171 | 0.672817523 | 0.488365472 | 0.509749577 | 0.711395 |
| TCGA-BJ-A0ZA-01A | 0.529099393 | 0.447768111 | 0.643042304 | 0.496355919 | 0.465109793 | 0.705155 |
| TCGA-BJ-A0ZB-01A | 0.54353106 | 0.442174837 | 0.643955253 | 0.486111421 | 0.471907884 | 0.6988296 |
| TCGA-BJ-A0ZC-01A | 0.532656216 | 0.429813068 | 0.633214099 | 0.504113811 | 0.437410305 | 0.7148797 |
| TCGA-BJ-A0ZE-01A | 0.520516675 | 0.421138297 | 0.627608294 | 0.498080744 | 0.438556808 | 0.698247 |
| TCGA-BJ-A0ZG-01A | 0.511334945 | 0.409656657 | 0.636296757 | 0.484730947 | 0.451975788 | 0.6898874 |
| TCGA-BJ-A0ZH-01A | 0.540479739 | 0.458757589 | 0.649558655 | 0.48219445 | 0.489956631 | 0.7084494 |
| TCGA-BJ-A0ZJ-01A | 0.55098547 | 0.510313091 | 0.654417123 | 0.480967556 | 0.510647236 | 0.6769149 |
| TCGA-BJ-A18Y-01A | 0.472847544 | 0.426796537 | 0.627947429 | 0.494967685 | 0.43788781 | 0.6825711 |
| TCGA-BJ-A18Z-01A | 0.551514607 | 0.470419143 | 0.65231907 | 0.479688814 | 0.498999773 | 0.6917384 |
| TCGA-BJ-A190-01A | 0.450961795 | 0.370590262 | 0.616512712 | 0.511572509 | 0.41396835 | 0.7062629 |
| TCGA-BJ-A191-01A | 0.543236139 | 0.478233984 | 0.638159076 | 0.49111663 | 0.449945036 | 0.6650927 |
| TCGA-BJ-A28R-01A | 0.575485158 | 0.471519578 | 0.66576184 | 0.487244387 | 0.499973666 | 0.6970091 |
| TCGA-BJ-A28S-01A | 0.453719443 | 0.407306287 | 0.610156514 | 0.493084133 | 0.428034654 | 0.6749572 |
| TCGA-BJ-A28T-01A | 0.601092251 | 0.535028712 | 0.682916217 | 0.485951688 | 0.546708836 | 0.6924686 |
| TCGA-BJ-A28V-01A | 0.469640279 | 0.384015256 | 0.62718038 | 0.50176574 | 0.424378262 | 0.7079414 |
| TCGA-BJ-A28W-01A | 0.588899798 | 0.603021399 | 0.661334982 | 0.476850319 | 0.541957419 | 0.6841538 |
| TCGA-BJ-A28X-01A | 0.588088198 | 0.550249976 | 0.682621561 | 0.487474077 | 0.548831501 | 0.6818792 |
| TCGA-BJ-A28Z-01A | 0.604004207 | 0.572448893 | 0.682890983 | 0.486467548 | 0.555623685 | 0.6448269 |
| TCGA-BJ-A290-01A | 0.479799075 | 0.455803832 | 0.629631364 | 0.478630766 | 0.443735671 | 0.6700834 |
| TCGA-BJ-A291-01A | 0.542985721 | 0.427696391 | 0.633079676 | 0.497778405 | 0.437796232 | 0.7153878 |
| TCGA-BJ-A2N7-01A | 0.482243455 | 0.408890618 | 0.624158209 | 0.492649537 | 0.439542431 | 0.6899803 |
| TCGA-BJ-A2N8-01A | 0.54796288 | 0.459086774 | 0.637431467 | 0.505117644 | 0.46214337 | 0.7024579 |
| TCGA-BJ-A2N9-01A | 0.525090341 | 0.507685157 | 0.619947644 | 0.50201079 | 0.485401861 | 0.7150245 |
| TCGA-BJ-A2NA-01A | 0.548544362 | 0.467025136 | 0.660981436 | 0.48477506 | 0.495181499 | 0.6882578 |
| TCGA-BJ-A2P4-01A | 0.483770952 | 0.445777502 | 0.625781898 | 0.486881625 | 0.457856287 | 0.6726883 |
| TCGA-BJ-A3EZ-01A | 0.545421469 | 0.461821543 | 0.655547489 | 0.492419135 | 0.48686089 | 0.70669 |
| TCGA-BJ-A3F0-01A | 0.502012622 | 0.420698629 | 0.643167265 | 0.493765533 | 0.451255328 | 0.7020419 |
| TCGA-BJ-A3PR-01A | 0.643139029 | 0.597836188 | 0.684773486 | 0.490886583 | 0.564402816 | 0.6893821 |
| TCGA-BJ-A3PT-01A | 0.517310046 | 0.468414889 | 0.640746666 | 0.490289932 | 0.501628019 | 0.6847657 |
| TCGA-BJ-A3PU-01A | 0.56588541 | 0.477128637 | 0.648531416 | 0.492840046 | 0.504415543 | 0.7059259 |
| TCGA-BJ-A45C-01A | 0.456001954 | 0.397500392 | 0.61033289 | 0.48445225 | 0.413200512 | 0.6754078 |
| TCGA-BJ-A45D-01A | 0.555136253 | 0.483395671 | 0.641536155 | 0.490906223 | 0.482148111 | 0.6672771 |
| TCGA-BJ-A45E-01A | 0.633038908 | 0.626428457 | 0.691565638 | 0.491055297 | 0.568554625 | 0.7117951 |
| TCGA-BJ-A45F-01A | 0.51575714 | 0.436460584 | 0.628546235 | 0.502401206 | 0.44870088 | 0.6982729 |
| TCGA-BJ-A45G-01A | 0.518042999 | 0.417935043 | 0.635863536 | 0.499555272 | 0.448760838 | 0.6910413 |
| TCGA-BJ-A45H-01A | 0.526921182 | 0.480094242 | 0.645358139 | 0.487007828 | 0.490772363 | 0.6853043 |
| TCGA-BJ-A45I-01A | 0.59690351 | 0.514030277 | 0.666404346 | 0.48899394 | 0.52994359 | 0.6895471 |
| TCGA-BJ-A45J-01A | 0.543021328 | 0.48540816 | 0.649995581 | 0.484185881 | 0.490778425 | 0.6674531 |
| TCGA-BJ-A45K-01A | 0.482339621 | 0.399154281 | 0.630140679 | 0.49150608 | 0.431021853 | 0.6842828 |
| TCGA-BJ-A4O8-01A | 0.540763843 | 0.487104193 | 0.658474297 | 0.479577733 | 0.511960455 | 0.659867 |
| TCGA-BJ-A4O9-01A | 0.493270493 | 0.413309658 | 0.625860889 | 0.481309388 | 0.438724163 | 0.6973638 |
| TCGA-CE-A13K-01A | 0.554320016 | 0.443029591 | 0.646801925 | 0.487461925 | 0.47536406 | 0.6754373 |
| TCGA-CE-A27D-01A | 0.58303631 | 0.521048127 | 0.664365337 | 0.479753809 | 0.522205941 | 0.6760542 |
| TCGA-CE-A3MD-01A | 0.562914706 | 0.49034129 | 0.645704458 | 0.487181758 | 0.469806059 | 0.6719358 |
| TCGA-CE-A3ME-01A | 0.523889572 | 0.450609374 | 0.649645228 | 0.481257613 | 0.475682075 | 0.6928508 |
| TCGA-CE-A481-01A | 0.560146594 | 0.509814217 | 0.652265888 | 0.494165082 | 0.487441516 | 0.6809593 |
| TCGA-CE-A482-01A | 0.553014056 | 0.470145606 | 0.64500094 | 0.495154925 | 0.471341888 | 0.6736574 |
| TCGA-CE-A483-01A | 0.514937317 | 0.440931764 | 0.643537746 | 0.497819812 | 0.464766195 | 0.7061095 |
| TCGA-CE-A484-01A | 0.467797048 | 0.469821283 | 0.6070037 | 0.493174905 | 0.437779779 | 0.68812 |
| TCGA-CE-A485-01A | 0.554265747 | 0.488599963 | 0.65702935 | 0.490431664 | 0.50132085 | 0.6693026 |
| TCGA-DE-A0XZ-01A | 0.562326208 | 0.521601405 | 0.653018949 | 0.493507856 | 0.501418036 | 0.6691062 |
| TCGA-DE-A0Y2-01A | 0.528010116 | 0.435836076 | 0.668923122 | 0.489892142 | 0.487417515 | 0.6928643 |
| TCGA-DE-A0Y3-01B | 0.526797748 | 0.419023733 | 0.65588956 | 0.485088933 | 0.470563103 | 0.6970096 |
| TCGA-DE-A2OL-01A | 0.490850235 | 0.392183826 | 0.631437342 | 0.49738707 | 0.429267699 | 0.6916429 |
| TCGA-DE-A3KN-01A | 0.519117201 | 0.452042527 | 0.648367084 | 0.496005932 | 0.468131949 | 0.65097 |
| TCGA-DE-A4M8-01A | 0.563139817 | 0.570558581 | 0.669206272 | 0.494038206 | 0.536110603 | 0.6710691 |
| TCGA-DE-A4M9-01A | 0.507236028 | 0.408003729 | 0.637117877 | 0.500136213 | 0.445734275 | 0.6937596 |
| TCGA-DE-A4MA-01A | 0.529434514 | 0.482522164 | 0.64057985 | 0.475577453 | 0.497002624 | 0.7113634 |
| TCGA-DE-A4MB-01A | 0.55643957 | 0.485240204 | 0.663842119 | 0.485216503 | 0.5240264 | 0.7022816 |
| TCGA-DE-A4MC-01A | 0.563830825 | 0.499239308 | 0.662274993 | 0.495514385 | 0.512796608 | 0.6965292 |
| TCGA-DE-A4MD-01A | 0.413921065 | 0.440521472 | 0.619931452 | 0.497616339 | 0.458093392 | 0.6983055 |
| TCGA-DE-A69J-01A | 0.524149001 | 0.454276501 | 0.656028518 | 0.488176317 | 0.489633959 | 0.6867987 |
| TCGA-DE-A69K-01A | 0.559822965 | 0.46918552 | 0.653471706 | 0.47934705 | 0.480756203 | 0.6631983 |
| TCGA-DE-A7U5-01A | 0.554406609 | 0.497159418 | 0.677257298 | 0.48758558 | 0.543530006 | 0.6817101 |
| TCGA-DJ-A13L-01A | 0.592058464 | 0.545887824 | 0.66223579 | 0.483520081 | 0.544076472 | 0.7106206 |
| TCGA-DJ-A13M-01A | 0.529007555 | 0.447065489 | 0.633194302 | 0.497358048 | 0.43731059 | 0.6895343 |
| TCGA-DJ-A13O-01A | 0.531812957 | 0.490770761 | 0.642134314 | 0.487977284 | 0.50671252 | 0.7022307 |
| TCGA-DJ-A13P-01A | 0.457473223 | 0.449353883 | 0.629488802 | 0.481137857 | 0.457345555 | 0.681279 |
| TCGA-DJ-A13R-01A | 0.544024302 | 0.592909126 | 0.655160443 | 0.491791305 | 0.523708657 | 0.6944216 |
| TCGA-DJ-A13S-01A | 0.528962225 | 0.412170811 | 0.63540236 | 0.507181797 | 0.440062038 | 0.6770184 |
| TCGA-DJ-A13T-01A | 0.520804378 | 0.437259518 | 0.652969071 | 0.492270843 | 0.464296737 | 0.6907588 |
| TCGA-DJ-A13U-01A | 0.498143207 | 0.420639828 | 0.64654441 | 0.487087596 | 0.465757251 | 0.6962887 |
| TCGA-DJ-A13V-01A | 0.511081465 | 0.464534541 | 0.639824835 | 0.477249049 | 0.466918768 | 0.6732075 |
| TCGA-DJ-A13W-01A | 0.460820187 | 0.450427242 | 0.625669625 | 0.456378547 | 0.459256972 | 0.6355368 |
| TCGA-DJ-A13X-01A | 0.495053701 | 0.434850133 | 0.639848233 | 0.482091699 | 0.463436542 | 0.6830608 |
| TCGA-DJ-A1QD-01A | 0.51320757 | 0.42588013 | 0.660290336 | 0.484163963 | 0.483707804 | 0.7084828 |
| TCGA-DJ-A1QE-01A | 0.547054217 | 0.423888804 | 0.668382657 | 0.484855919 | 0.499145205 | 0.6908834 |
| TCGA-DJ-A1QF-01A | 0.515295014 | 0.422093643 | 0.645690634 | 0.474956818 | 0.462738585 | 0.6893312 |
| TCGA-DJ-A1QG-01A | 0.508831405 | 0.449939558 | 0.644784081 | 0.499293705 | 0.45561925 | 0.6986704 |
| TCGA-DJ-A1QH-01A | 0.530731392 | 0.449566005 | 0.654443982 | 0.483433932 | 0.485255935 | 0.6983307 |
| TCGA-DJ-A1QI-01A | 0.533569993 | 0.449521352 | 0.654062645 | 0.48195106 | 0.479615971 | 0.6873534 |
| TCGA-DJ-A1QL-01A | 0.503566674 | 0.42936792 | 0.627668962 | 0.489760442 | 0.440397357 | 0.6749259 |
| TCGA-DJ-A1QM-01A | 0.500431251 | 0.416395505 | 0.642161804 | 0.487435476 | 0.482769607 | 0.6989933 |
| TCGA-DJ-A1QN-01A | 0.543747171 | 0.452983242 | 0.648449057 | 0.486025207 | 0.486139304 | 0.6645719 |
| TCGA-DJ-A1QO-01A | 0.52280737 | 0.457389941 | 0.658252417 | 0.48273457 | 0.505506727 | 0.7228459 |
| TCGA-DJ-A1QQ-01A | 0.528606942 | 0.435496978 | 0.656576706 | 0.484536403 | 0.48142097 | 0.6598394 |
| TCGA-DJ-A2PN-01A | 0.501293447 | 0.426383314 | 0.656953178 | 0.488166164 | 0.47447309 | 0.6989403 |
| TCGA-DJ-A2PO-01A | 0.526997772 | 0.49474037 | 0.649311698 | 0.484358028 | 0.504029141 | 0.6790938 |
| TCGA-DJ-A2PP-01A | 0.505190762 | 0.399364494 | 0.635510211 | 0.501762175 | 0.432089515 | 0.7347609 |
| TCGA-DJ-A2PQ-01A | 0.475825456 | 0.425768823 | 0.630975633 | 0.491990897 | 0.454016475 | 0.6957841 |
| TCGA-DJ-A2PR-01A | 0.567039448 | 0.50007472 | 0.659918292 | 0.487948034 | 0.491642243 | 0.6735405 |
| TCGA-DJ-A2PS-01A | 0.52976523 | 0.446948599 | 0.651818139 | 0.49075204 | 0.485926711 | 0.6949967 |
| TCGA-DJ-A2PT-01A | 0.565102549 | 0.529304143 | 0.664627403 | 0.486406918 | 0.539417024 | 0.698788 |
| TCGA-DJ-A2PU-01A | 0.474067078 | 0.443265268 | 0.646080107 | 0.47447105 | 0.475988703 | 0.672225 |
| TCGA-DJ-A2PV-01A | 0.485036503 | 0.451452428 | 0.652077949 | 0.478591064 | 0.476478467 | 0.6660199 |
| TCGA-DJ-A2PW-01A | 0.540647678 | 0.482091022 | 0.64955565 | 0.478582157 | 0.495477885 | 0.6826111 |
| TCGA-DJ-A2PX-01A | 0.547142601 | 0.53378742 | 0.65700851 | 0.480204844 | 0.508396282 | 0.6869072 |
| TCGA-DJ-A2PY-01A | 0.518387202 | 0.457207967 | 0.660237334 | 0.482181123 | 0.498865683 | 0.7044333 |
| TCGA-DJ-A2PZ-01A | 0.528210101 | 0.446575338 | 0.646205438 | 0.48509357 | 0.489407462 | 0.7007356 |
| TCGA-DJ-A2Q0-01A | 0.465844974 | 0.403001185 | 0.630445443 | 0.512297322 | 0.436768399 | 0.7624008 |
| TCGA-DJ-A2Q1-01A | 0.515865608 | 0.518077713 | 0.667635533 | 0.479643192 | 0.550966438 | 0.6914812 |
| TCGA-DJ-A2Q2-01A | 0.497326525 | 0.441356339 | 0.643353456 | 0.501205608 | 0.474060616 | 0.7134646 |
| TCGA-DJ-A2Q3-01A | 0.571391767 | 0.494713164 | 0.668562044 | 0.487526642 | 0.52343771 | 0.6975255 |
| TCGA-DJ-A2Q4-01A | 0.548911957 | 0.473492753 | 0.658415421 | 0.482908329 | 0.490967199 | 0.6754906 |
| TCGA-DJ-A2Q5-01A | 0.537669006 | 0.530174714 | 0.665900222 | 0.483816154 | 0.519288857 | 0.6654352 |
| TCGA-DJ-A2Q6-01A | 0.526429557 | 0.452754832 | 0.65588509 | 0.487652039 | 0.485932467 | 0.6938633 |
| TCGA-DJ-A2Q7-01A | 0.500937666 | 0.472565644 | 0.641059074 | 0.485400446 | 0.478390615 | 0.6779869 |
| TCGA-DJ-A2Q9-01A | 0.543686243 | 0.545829548 | 0.697090803 | 0.473452548 | 0.558432769 | 0.7127583 |
| TCGA-DJ-A2QA-01A | 0.500000167 | 0.458867336 | 0.617217794 | 0.472058057 | 0.462324818 | 0.6650085 |
| TCGA-DJ-A2QB-01A | 0.480409504 | 0.431657079 | 0.63930863 | 0.511956242 | 0.444569626 | 0.7289986 |
| TCGA-DJ-A2QC-01A | 0.526608687 | 0.482731089 | 0.629932918 | 0.472025029 | 0.467547763 | 0.6753582 |
| TCGA-DJ-A3UK-01A | 0.500965937 | 0.463532449 | 0.633020806 | 0.482851412 | 0.460268433 | 0.6629202 |
| TCGA-DJ-A3UM-01A | 0.526578439 | 0.45745315 | 0.646534667 | 0.477704014 | 0.486205211 | 0.6797095 |
| TCGA-DJ-A3UN-01A | 0.513594459 | 0.46955023 | 0.634696606 | 0.496893328 | 0.466685034 | 0.6636442 |
| TCGA-DJ-A3UO-01A | 0.532510099 | 0.448591223 | 0.640258107 | 0.486305742 | 0.465923775 | 0.641162 |
| TCGA-DJ-A3UP-01A | 0.507655578 | 0.461735309 | 0.637917857 | 0.507955124 | 0.45619113 | 0.6727991 |
| TCGA-DJ-A3UQ-01A | 0.540208967 | 0.457669343 | 0.659981094 | 0.483207885 | 0.51338512 | 0.6775193 |
| TCGA-DJ-A3UR-01A | 0.485164356 | 0.43221355 | 0.62857413 | 0.4739635 | 0.455703199 | 0.6743856 |
| TCGA-DJ-A3US-01A | 0.557498248 | 0.520253919 | 0.656492975 | 0.490283225 | 0.513293086 | 0.6900671 |
| TCGA-DJ-A3UT-01A | 0.462992122 | 0.407077348 | 0.621349325 | 0.495181664 | 0.428110049 | 0.7030107 |
| TCGA-DJ-A3UU-01A | 0.512704018 | 0.471087789 | 0.647183645 | 0.485084971 | 0.493711278 | 0.6711947 |
| TCGA-DJ-A3UV-01A | 0.514666834 | 0.464064565 | 0.64415636 | 0.493870877 | 0.480963136 | 0.664713 |
| TCGA-DJ-A3UW-01A | 0.546728192 | 0.53064948 | 0.666213568 | 0.485223891 | 0.538113437 | 0.6743006 |
| TCGA-DJ-A3UX-01A | 0.515973797 | 0.461776633 | 0.645454231 | 0.493517601 | 0.489295217 | 0.6842274 |
| TCGA-DJ-A3UY-01A | 0.528354246 | 0.446720051 | 0.658237305 | 0.483252191 | 0.507608307 | 0.6992073 |
| TCGA-DJ-A3UZ-01A | 0.513675301 | 0.462619969 | 0.643185726 | 0.494281812 | 0.477527262 | 0.6855552 |
| TCGA-DJ-A3V0-01A | 0.508635054 | 0.472738341 | 0.641782455 | 0.480524844 | 0.491473258 | 0.6849124 |
| TCGA-DJ-A3V2-01A | 0.553886333 | 0.558274184 | 0.650230791 | 0.488280792 | 0.530468317 | 0.6873261 |
| TCGA-DJ-A3V3-01A | 0.565757423 | 0.506488972 | 0.644405417 | 0.486510857 | 0.492265093 | 0.680307 |
| TCGA-DJ-A3V4-01A | 0.51492817 | 0.441316967 | 0.645308574 | 0.488676794 | 0.480557892 | 0.7063772 |
| TCGA-DJ-A3V5-01A | 0.531992739 | 0.429196726 | 0.643472246 | 0.490498875 | 0.491158057 | 0.6942283 |
| TCGA-DJ-A3V6-01A | 0.542173961 | 0.463454422 | 0.649601431 | 0.49127424 | 0.493444145 | 0.710097 |
| TCGA-DJ-A3V7-01A | 0.541319535 | 0.469818754 | 0.645205222 | 0.489645715 | 0.485249868 | 0.7064891 |
| TCGA-DJ-A3V8-01A | 0.518052281 | 0.434983617 | 0.65020697 | 0.488926841 | 0.481421342 | 0.7076502 |
| TCGA-DJ-A3V9-01A | 0.549617512 | 0.458635402 | 0.652244055 | 0.488404649 | 0.481680449 | 0.677381 |
| TCGA-DJ-A3VA-01A | 0.560181804 | 0.466484933 | 0.652792111 | 0.495645991 | 0.473761098 | 0.6883135 |
| TCGA-DJ-A3VB-01A | 0.553852473 | 0.490282783 | 0.653520414 | 0.484636977 | 0.516620455 | 0.7088578 |
| TCGA-DJ-A3VD-01A | 0.561479706 | 0.536846723 | 0.685522176 | 0.481479611 | 0.549901473 | 0.672384 |
| TCGA-DJ-A3VE-01A | 0.546811078 | 0.437978033 | 0.649382999 | 0.484460116 | 0.481473126 | 0.685812 |
| TCGA-DJ-A3VF-01A | 0.553677499 | 0.45671881 | 0.674682017 | 0.488195812 | 0.540831363 | 0.6740274 |
| TCGA-DJ-A3VG-01A | 0.503373077 | 0.435631856 | 0.62635809 | 0.495188155 | 0.436385623 | 0.7020561 |
| TCGA-DJ-A3VI-01A | 0.552114818 | 0.44668919 | 0.65663567 | 0.494246168 | 0.477788045 | 0.7037408 |
| TCGA-DJ-A3VK-01A | 0.52248386 | 0.458673231 | 0.623261824 | 0.503300839 | 0.450404583 | 0.6859712 |
| TCGA-DJ-A3VL-01A | 0.521796702 | 0.540144102 | 0.631333439 | 0.50314172 | 0.488092057 | 0.6829115 |
| TCGA-DJ-A3VM-01A | 0.514228897 | 0.422440049 | 0.632955703 | 0.503245752 | 0.44130751 | 0.6805526 |
| TCGA-DJ-A4UL-01A | 0.501112858 | 0.509785555 | 0.635244045 | 0.484623095 | 0.475617048 | 0.6720309 |
| TCGA-DJ-A4UP-01A | 0.53731183 | 0.502434106 | 0.668555208 | 0.490076422 | 0.508396401 | 0.6951526 |
| TCGA-DJ-A4UR-01A | 0.510019132 | 0.489877841 | 0.625768328 | 0.491340987 | 0.458853 | 0.6750655 |
| TCGA-DJ-A4UT-01A | 0.595016235 | 0.536315725 | 0.672029789 | 0.488057527 | 0.528681218 | 0.6765686 |
| TCGA-DJ-A4UW-01A | 0.505717519 | 0.447177062 | 0.640421877 | 0.474882676 | 0.484207817 | 0.6828172 |
| TCGA-DJ-A4V0-01A | 0.566520697 | 0.512718057 | 0.660940555 | 0.501637885 | 0.513401772 | 0.6978419 |
| TCGA-DJ-A4V2-01A | 0.519075393 | 0.457910546 | 0.665240908 | 0.489542272 | 0.507720363 | 0.689995 |
| TCGA-DJ-A4V4-01A | 0.517704705 | 0.426379217 | 0.636475418 | 0.478071573 | 0.471254716 | 0.7016211 |
| TCGA-DJ-A4V5-01A | 0.554997857 | 0.500712651 | 0.668371962 | 0.484013773 | 0.527304588 | 0.6659246 |
| TCGA-DO-A1JZ-01A | 0.482332499 | 0.442235235 | 0.636614756 | 0.475814363 | 0.437217003 | 0.6553241 |
| TCGA-DO-A1K0-01A | 0.517614085 | 0.416823454 | 0.655287642 | 0.483201769 | 0.474887843 | 0.6826053 |
| TCGA-DO-A2HM-01B | 0.629166227 | 0.600430495 | 0.697135761 | 0.492673023 | 0.586302702 | 0.6958957 |
| TCGA-E3-A3DY-01A | 0.509320235 | 0.474524217 | 0.645502898 | 0.479994108 | 0.483668846 | 0.6849087 |
| TCGA-E3-A3DZ-01A | 0.504204904 | 0.433234971 | 0.645201882 | 0.505559836 | 0.433547139 | 0.7004757 |
| TCGA-E3-A3E0-01A | 0.509655917 | 0.482643954 | 0.64141368 | 0.493181889 | 0.46986384 | 0.7033698 |
| TCGA-E3-A3E1-01A | 0.516289923 | 0.437827301 | 0.648023078 | 0.478202381 | 0.482232627 | 0.6909388 |
| TCGA-E3-A3E2-01A | 0.525450069 | 0.510630617 | 0.6491045 | 0.472939516 | 0.505356031 | 0.6749104 |
| TCGA-E3-A3E3-01A | 0.522636244 | 0.431188363 | 0.644772609 | 0.482143364 | 0.47473319 | 0.7006142 |
| TCGA-E3-A3E5-01A | 0.505030646 | 0.445711786 | 0.634817883 | 0.489351726 | 0.454819623 | 0.6888915 |
| TCGA-E8-A242-01A | 0.583576234 | 0.510923074 | 0.668017658 | 0.483111049 | 0.537796192 | 0.7097262 |
| TCGA-E8-A2EA-01A | 0.582717561 | 0.569545982 | 0.662514114 | 0.496102645 | 0.547203865 | 0.6951091 |
| TCGA-E8-A2JQ-01A | 0.587593523 | 0.580940258 | 0.67359563 | 0.49870975 | 0.558046743 | 0.6695333 |
| TCGA-E8-A3X7-01A | 0.561265805 | 0.466557423 | 0.640599754 | 0.483334023 | 0.489332701 | 0.7016076 |
| TCGA-E8-A413-01A | 0.549771546 | 0.459498895 | 0.65963193 | 0.480362304 | 0.511547276 | 0.6851609 |
| TCGA-E8-A414-01A | 0.569724097 | 0.463036571 | 0.665885276 | 0.485248911 | 0.519295688 | 0.6836776 |
| TCGA-E8-A415-01A | 0.541192808 | 0.447750514 | 0.65101179 | 0.490884007 | 0.481622468 | 0.701382 |
| TCGA-E8-A416-01A | 0.507922529 | 0.406073673 | 0.624901716 | 0.504885261 | 0.453205007 | 0.7206093 |
| TCGA-E8-A417-01A | 0.567025254 | 0.496694955 | 0.649746524 | 0.499792977 | 0.492066491 | 0.7043823 |
| TCGA-E8-A418-01A | 0.570907503 | 0.468782376 | 0.651931824 | 0.50180689 | 0.493128186 | 0.741619 |
| TCGA-E8-A419-01A | 0.624406005 | 0.552309595 | 0.68011406 | 0.493738968 | 0.54589151 | 0.7039696 |
| TCGA-E8-A432-01A | 0.563573266 | 0.48094286 | 0.645300201 | 0.489240608 | 0.479258442 | 0.6735298 |
| TCGA-E8-A433-01A | 0.537602475 | 0.476016665 | 0.642739146 | 0.481303291 | 0.505385664 | 0.725342 |
| TCGA-E8-A434-01A | 0.540072321 | 0.437954824 | 0.644383727 | 0.49173277 | 0.474207639 | 0.7101624 |
| TCGA-E8-A436-01A | 0.57167832 | 0.476061337 | 0.661562416 | 0.495345241 | 0.515510836 | 0.6923076 |
| TCGA-E8-A437-01A | 0.501847813 | 0.451875734 | 0.634736501 | 0.476736079 | 0.467797323 | 0.66672 |
| TCGA-E8-A438-01A | 0.537204433 | 0.4831012 | 0.633977328 | 0.479897403 | 0.453986487 | 0.6570445 |
| TCGA-E8-A44K-01A | 0.559501275 | 0.478806664 | 0.656441112 | 0.483478845 | 0.500849112 | 0.705597 |
| TCGA-E8-A44M-01A | 0.631877636 | 0.575883503 | 0.662719943 | 0.500847315 | 0.535994551 | 0.6685902 |
| TCGA-EL-A3CL-01A | 0.536242996 | 0.459570263 | 0.663667163 | 0.49728743 | 0.47581098 | 0.6762484 |
| TCGA-EL-A3CM-01A | 0.509967589 | 0.444565233 | 0.648089386 | 0.488553708 | 0.491768041 | 0.6845175 |
| TCGA-EL-A3CN-01A | 0.582759943 | 0.506190863 | 0.673186326 | 0.485040123 | 0.534829111 | 0.6707374 |
| TCGA-EL-A3CO-01A | 0.52242807 | 0.491453051 | 0.675902205 | 0.497400593 | 0.548007768 | 0.6889872 |
| TCGA-EL-A3CP-01A | 0.495258874 | 0.412079024 | 0.64498334 | 0.484802809 | 0.458352185 | 0.6925022 |
| TCGA-EL-A3CR-01A | 0.529740577 | 0.513050433 | 0.647285202 | 0.476547988 | 0.526560923 | 0.6801854 |
| TCGA-EL-A3CS-01A | 0.456666129 | 0.402424437 | 0.639883903 | 0.486423212 | 0.460806414 | 0.6942981 |
| TCGA-EL-A3CT-01A | 0.521905803 | 0.483717727 | 0.662231105 | 0.490875437 | 0.507796718 | 0.7054455 |
| TCGA-EL-A3CU-01A | 0.552137809 | 0.481147324 | 0.669107954 | 0.480701794 | 0.515904341 | 0.6965595 |
| TCGA-EL-A3CV-01A | 0.551125877 | 0.534729557 | 0.693446112 | 0.477158795 | 0.560238755 | 0.675386 |
| TCGA-EL-A3CW-01A | 0.538691194 | 0.482668831 | 0.658163088 | 0.486453676 | 0.517688313 | 0.7137066 |
| TCGA-EL-A3CX-01A | 0.520743157 | 0.457627139 | 0.628861849 | 0.478438963 | 0.461194498 | 0.6619591 |
| TCGA-EL-A3CY-01A | 0.569816292 | 0.57833944 | 0.710749782 | 0.484264135 | 0.58977069 | 0.6681838 |
| TCGA-EL-A3CZ-01A | 0.499726028 | 0.413607359 | 0.634969073 | 0.500523756 | 0.447903912 | 0.7054843 |
| TCGA-EL-A3D0-01A | 0.48828329 | 0.461941147 | 0.633332265 | 0.483773587 | 0.458255628 | 0.6902465 |
| TCGA-EL-A3D1-01A | 0.542717525 | 0.483755392 | 0.6413453 | 0.473124984 | 0.495188337 | 0.63882 |
| TCGA-EL-A3D4-01A | 0.500078985 | 0.433985175 | 0.647300816 | 0.48893831 | 0.450291168 | 0.6600963 |
| TCGA-EL-A3D5-01A | 0.481013435 | 0.407765479 | 0.624814427 | 0.51002431 | 0.429566287 | 0.7109929 |
| TCGA-EL-A3D6-01A | 0.523979747 | 0.488382052 | 0.660949081 | 0.491561752 | 0.524240388 | 0.7030701 |
| TCGA-EL-A3GO-01A | 0.507565295 | 0.449488202 | 0.651570175 | 0.507202363 | 0.4580337 | 0.7200069 |
| TCGA-EL-A3GP-01A | 0.500619265 | 0.510067506 | 0.651964675 | 0.496723857 | 0.5228355 | 0.6734126 |
| TCGA-EL-A3GQ-01A | 0.493005507 | 0.457632694 | 0.648876161 | 0.48689363 | 0.477392099 | 0.6988608 |
| TCGA-EL-A3GR-01A | 0.529181089 | 0.429398752 | 0.667839217 | 0.490648051 | 0.504017174 | 0.6770913 |
| TCGA-EL-A3GS-01A | 0.569356343 | 0.426881751 | 0.673126473 | 0.493042879 | 0.48392253 | 0.7126717 |
| TCGA-EL-A3GU-01A | 0.527444226 | 0.433540191 | 0.662390044 | 0.480708838 | 0.49490295 | 0.7026016 |
| TCGA-EL-A3GV-01A | 0.540344506 | 0.452315824 | 0.665311833 | 0.479013799 | 0.507428899 | 0.6819785 |
| TCGA-EL-A3GW-01A | 0.550457322 | 0.422597665 | 0.64781443 | 0.497085986 | 0.459586068 | 0.6711842 |
| TCGA-EL-A3GX-01A | 0.551667132 | 0.492661715 | 0.668543016 | 0.485001369 | 0.521743702 | 0.6983514 |
| TCGA-EL-A3GY-01A | 0.503623904 | 0.464181636 | 0.665343293 | 0.485526001 | 0.513076487 | 0.6907885 |
| TCGA-EL-A3GZ-01A | 0.531865887 | 0.451360507 | 0.646479228 | 0.482119403 | 0.482432704 | 0.6612336 |
| TCGA-EL-A3H1-01A | 0.495471527 | 0.417001717 | 0.634867348 | 0.495260643 | 0.44589039 | 0.7127129 |
| TCGA-EL-A3H2-01A | 0.498455377 | 0.471474458 | 0.644182619 | 0.491363914 | 0.443999106 | 0.7224189 |
| TCGA-EL-A3H3-01A | 0.576599965 | 0.525145351 | 0.653405659 | 0.482426862 | 0.51287883 | 0.6384395 |
| TCGA-EL-A3H4-01A | 0.492538965 | 0.424569321 | 0.664941645 | 0.486082857 | 0.48242072 | 0.6844374 |
| TCGA-EL-A3H5-01A | 0.539148506 | 0.519659833 | 0.661394681 | 0.459443165 | 0.528154571 | 0.693528 |
| TCGA-EL-A3H7-01A | 0.55787017 | 0.49310329 | 0.668124823 | 0.489528639 | 0.528896221 | 0.6937566 |
| TCGA-EL-A3H8-01A | 0.498550995 | 0.424666644 | 0.651081784 | 0.482629304 | 0.47479996 | 0.6859652 |
| TCGA-EL-A3MW-01A | 0.498313535 | 0.470595444 | 0.634083478 | 0.48458103 | 0.470611814 | 0.6881854 |
| TCGA-EL-A3MX-01A | 0.425602052 | 0.457961851 | 0.604010831 | 0.465211796 | 0.447992098 | 0.6868746 |
| TCGA-EL-A3MY-01A | 0.51333762 | 0.465081946 | 0.671730949 | 0.479605771 | 0.534157964 | 0.7116586 |
| TCGA-EL-A3MZ-01A | 0.544786967 | 0.464518605 | 0.650733617 | 0.485604932 | 0.492226651 | 0.728439 |
| TCGA-EL-A3N2-01A | 0.544999735 | 0.442311212 | 0.655724013 | 0.489674913 | 0.487583721 | 0.6854774 |
| TCGA-EL-A3N3-01A | 0.570151427 | 0.502374942 | 0.676312398 | 0.494229758 | 0.526296679 | 0.6949034 |
| TCGA-EL-A3T0-01A | 0.52899041 | 0.449990465 | 0.657239232 | 0.485837917 | 0.489146076 | 0.6717071 |
| TCGA-EL-A3T1-01A | 0.552924145 | 0.503727747 | 0.671292346 | 0.482440093 | 0.516147771 | 0.6740332 |
| TCGA-EL-A3T2-01A | 0.514359804 | 0.415855732 | 0.639607205 | 0.510164135 | 0.44118844 | 0.6971468 |
| TCGA-EL-A3T3-01A | 0.536031051 | 0.470836756 | 0.654433215 | 0.490071937 | 0.508459449 | 0.6793811 |
| TCGA-EL-A3T6-01A | 0.566000184 | 0.524773257 | 0.676761787 | 0.497243528 | 0.541588633 | 0.7042726 |
| TCGA-EL-A3T7-01A | 0.518263678 | 0.476137332 | 0.649722386 | 0.486906168 | 0.494223246 | 0.6764771 |
| TCGA-EL-A3T8-01A | 0.51406648 | 0.458559114 | 0.652042844 | 0.485361491 | 0.482419846 | 0.6603531 |
| TCGA-EL-A3T9-01A | 0.620154786 | 0.463507188 | 0.657070386 | 0.47200355 | 0.519674595 | 0.6773701 |
| TCGA-EL-A3TA-01A | 0.504932059 | 0.485633806 | 0.638764324 | 0.479377012 | 0.501427726 | 0.6739356 |
| TCGA-EL-A3TB-01A | 0.537998645 | 0.462131281 | 0.630876102 | 0.488787026 | 0.473633864 | 0.6764313 |
| TCGA-EL-A3ZG-01A | 0.495974459 | 0.429487337 | 0.630125313 | 0.486724248 | 0.452831313 | 0.7085648 |
| TCGA-EL-A3ZH-01A | 0.478805588 | 0.432193826 | 0.633870081 | 0.511335849 | 0.446619868 | 0.6979938 |
| TCGA-EL-A3ZK-01A | 0.592159464 | 0.484884894 | 0.678758206 | 0.499091782 | 0.517359709 | 0.6898918 |
| TCGA-EL-A3ZL-01A | 0.488326118 | 0.472067562 | 0.632860377 | 0.478429926 | 0.454578556 | 0.6726133 |
| TCGA-EL-A3ZM-01A | 0.556625179 | 0.510479682 | 0.6461364 | 0.49028552 | 0.512211249 | 0.7365365 |
| TCGA-EL-A3ZN-01A | 0.516168245 | 0.462117119 | 0.628344858 | 0.486926234 | 0.454981783 | 0.6732874 |
| TCGA-EL-A3ZO-01A | 0.59662463 | 0.533446762 | 0.654047876 | 0.488963223 | 0.546927094 | 0.7058745 |
| TCGA-EL-A3ZP-01A | 0.554829665 | 0.538711536 | 0.651651152 | 0.486785317 | 0.513975663 | 0.6883211 |
| TCGA-EL-A3ZQ-01A | 0.520058807 | 0.517310954 | 0.627401013 | 0.462458532 | 0.500363164 | 0.6654549 |
| TCGA-EL-A3ZR-01A | 0.496363864 | 0.425593493 | 0.617972828 | 0.498743939 | 0.426364046 | 0.6783974 |
| TCGA-EL-A3ZS-01A | 0.536427944 | 0.519515922 | 0.661708173 | 0.490042722 | 0.517219684 | 0.6878732 |
| TCGA-EL-A3ZT-01A | 0.533502158 | 0.460625083 | 0.650278239 | 0.489596292 | 0.480819672 | 0.6856403 |
| TCGA-EL-A4JV-01A | 0.50575649 | 0.41181144 | 0.621203495 | 0.49599018 | 0.4430411 | 0.6811947 |
| TCGA-EL-A4JW-01A | 0.534056069 | 0.432161933 | 0.667198811 | 0.485801598 | 0.492795471 | 0.6947737 |
| TCGA-EL-A4JX-01A | 0.654655523 | 0.580847703 | 0.689524923 | 0.497772446 | 0.570558072 | 0.6771735 |
| TCGA-EL-A4JZ-01A | 0.554420223 | 0.541298535 | 0.659876182 | 0.487155598 | 0.514072204 | 0.6701712 |
| TCGA-EL-A4K0-01A | 0.52788311 | 0.479633719 | 0.655610588 | 0.481392191 | 0.494513063 | 0.6706725 |
| TCGA-EL-A4K1-01A | 0.535117435 | 0.475384454 | 0.641200043 | 0.474967525 | 0.48679589 | 0.7157245 |
| TCGA-EL-A4K2-01A | 0.521008007 | 0.48871721 | 0.637537688 | 0.495549641 | 0.485515188 | 0.6964305 |
| TCGA-EL-A4K4-01A | 0.50126159 | 0.459357337 | 0.653360948 | 0.488251937 | 0.506364904 | 0.6852232 |
| TCGA-EL-A4K6-01A | 0.455451213 | 0.456743789 | 0.594055225 | 0.498348428 | 0.419207557 | 0.6469593 |
| TCGA-EL-A4K7-01A | 0.498763354 | 0.478769632 | 0.628476172 | 0.475082393 | 0.463911312 | 0.7059238 |
| TCGA-EL-A4K9-01A | 0.535388667 | 0.470179275 | 0.654173729 | 0.495741176 | 0.478761126 | 0.6704393 |
| TCGA-EL-A4KD-01A | 0.517303869 | 0.465857469 | 0.643426542 | 0.486010144 | 0.476934426 | 0.6756199 |
| TCGA-EL-A4KG-01A | 0.574072574 | 0.506016567 | 0.661209253 | 0.481342156 | 0.515810156 | 0.709338 |
| TCGA-EL-A4KH-01A | 0.508232838 | 0.474725474 | 0.625229892 | 0.469512616 | 0.47383112 | 0.6619658 |
| TCGA-EL-A4KI-01A | 0.470368903 | 0.455095042 | 0.606709829 | 0.50091521 | 0.424690164 | 0.6430873 |
| TCGA-EM-A1CS-01A | 0.475923798 | 0.43200072 | 0.637678354 | 0.483337592 | 0.459837436 | 0.6767047 |
| TCGA-EM-A1CT-01A | 0.516766053 | 0.4484092 | 0.645912082 | 0.484047644 | 0.480351271 | 0.6781642 |
| TCGA-EM-A1CU-01A | 0.510759116 | 0.464283763 | 0.654664635 | 0.481950628 | 0.488112856 | 0.6757777 |
| TCGA-EM-A1CV-01A | 0.473344058 | 0.442314465 | 0.636198725 | 0.48846989 | 0.449369574 | 0.6859206 |
| TCGA-EM-A1CW-01A | 0.526702518 | 0.476458417 | 0.64358341 | 0.497620853 | 0.478779676 | 0.7263394 |
| TCGA-EM-A1YA-01A | 0.506601239 | 0.382591248 | 0.638652595 | 0.497553789 | 0.442511829 | 0.7526161 |
| TCGA-EM-A1YB-01A | 0.449677768 | 0.430184449 | 0.626946189 | 0.500205115 | 0.448065876 | 0.7852777 |
| TCGA-EM-A1YC-01A | 0.449542866 | 0.390101045 | 0.628423637 | 0.494443449 | 0.439165357 | 0.7252651 |
| TCGA-EM-A1YD-01A | 0.505107218 | 0.411276221 | 0.630798837 | 0.499580931 | 0.458479445 | 0.698984 |
| TCGA-EM-A1YE-01A | 0.514886784 | 0.443733858 | 0.620558742 | 0.47223067 | 0.435950103 | 0.6968848 |
| TCGA-EM-A22I-01A | 0.529647896 | 0.441387692 | 0.663473324 | 0.485671474 | 0.494601941 | 0.6837457 |
| TCGA-EM-A22J-01A | 0.470643543 | 0.439498362 | 0.630137754 | 0.508143876 | 0.441710598 | 0.7246736 |
| TCGA-EM-A22K-01A | 0.481358596 | 0.437275576 | 0.629132091 | 0.497383353 | 0.445926693 | 0.6825325 |
| TCGA-EM-A22L-01A | 0.508488491 | 0.413113789 | 0.636843628 | 0.50538641 | 0.425195388 | 0.7055394 |
| TCGA-EM-A22M-01A | 0.616936652 | 0.598389829 | 0.67230648 | 0.486664213 | 0.535088823 | 0.6567375 |
| TCGA-EM-A22N-01A | 0.460610982 | 0.423656796 | 0.631842809 | 0.521213654 | 0.447528734 | 0.7071621 |
| TCGA-EM-A22O-01A | 0.513283754 | 0.438889246 | 0.632832316 | 0.486523955 | 0.465617243 | 0.7178672 |
| TCGA-EM-A22P-01A | 0.523974923 | 0.53204706 | 0.665911078 | 0.486030419 | 0.53419488 | 0.6684984 |
| TCGA-EM-A22Q-01A | 0.460997467 | 0.461351291 | 0.603932412 | 0.466713236 | 0.453029382 | 0.6634316 |
| TCGA-EM-A2CJ-01A | 0.465111658 | 0.418473345 | 0.623410068 | 0.50141636 | 0.438083481 | 0.705684 |
| TCGA-EM-A2CK-01A | 0.472831703 | 0.423719479 | 0.624910359 | 0.494196161 | 0.433733304 | 0.6929942 |
| TCGA-EM-A2CL-01A | 0.477811051 | 0.396186735 | 0.633871103 | 0.506141916 | 0.422112775 | 0.694312 |
| TCGA-EM-A2CN-01A | 0.44104478 | 0.416525889 | 0.617063572 | 0.486543344 | 0.434233722 | 0.6714018 |
| TCGA-EM-A2CO-01A | 0.469876045 | 0.406684622 | 0.627224128 | 0.50930566 | 0.436417367 | 0.7223733 |
| TCGA-EM-A2CP-01A | 0.61294922 | 0.580466313 | 0.679569769 | 0.514212556 | 0.53400237 | 0.6943774 |
| TCGA-EM-A2CQ-01A | 0.482369898 | 0.395866896 | 0.637082729 | 0.494635332 | 0.443499979 | 0.718541 |
| TCGA-EM-A2CR-01A | 0.45012446 | 0.469482761 | 0.603186744 | 0.475735169 | 0.457978898 | 0.6728562 |
| TCGA-EM-A2CS-01A | 0.479523292 | 0.439314601 | 0.645299784 | 0.495231053 | 0.452210766 | 0.6637435 |
| TCGA-EM-A2CT-01A | 0.500482578 | 0.421707312 | 0.64178314 | 0.494786072 | 0.437349104 | 0.7258244 |
| TCGA-EM-A2CU-01A | 0.540715752 | 0.498489159 | 0.651401324 | 0.482646174 | 0.482712588 | 0.6596682 |
| TCGA-EM-A2OV-01A | 0.485262014 | 0.396283637 | 0.639685665 | 0.503099425 | 0.434189101 | 0.7825414 |
| TCGA-EM-A2OW-01A | 0.48163297 | 0.437979589 | 0.641952454 | 0.507920186 | 0.453356606 | 0.7582204 |
| TCGA-EM-A2OX-01A | 0.536642023 | 0.44005436 | 0.641319141 | 0.486756744 | 0.475467521 | 0.658893 |
| TCGA-EM-A2OY-01A | 0.442763842 | 0.412134226 | 0.624756318 | 0.519302325 | 0.428037097 | 0.7453037 |
| TCGA-EM-A2OZ-01A | 0.523344474 | 0.441739117 | 0.649378859 | 0.488050196 | 0.474321834 | 0.7106142 |
| TCGA-EM-A2P0-01A | 0.537216105 | 0.437501978 | 0.65685178 | 0.489955864 | 0.480964129 | 0.6901832 |
| TCGA-EM-A2P1-01A | 0.578717944 | 0.56977314 | 0.686093792 | 0.491626908 | 0.550675535 | 0.7053047 |
| TCGA-EM-A2P2-01A | 0.517267638 | 0.415216534 | 0.639258475 | 0.51463068 | 0.444625009 | 0.6643728 |
| TCGA-EM-A2P3-01A | 0.527447473 | 0.442908866 | 0.64999933 | 0.477866918 | 0.496503463 | 0.6938642 |
| TCGA-EM-A3AI-01A | 0.475675894 | 0.428136982 | 0.606864947 | 0.491891436 | 0.427876241 | 0.7096852 |
| TCGA-EM-A3AJ-01A | 0.62109591 | 0.53076267 | 0.664463212 | 0.508469312 | 0.517624728 | 0.7158468 |
| TCGA-EM-A3AK-01A | 0.546094742 | 0.450570129 | 0.640003587 | 0.486076537 | 0.476436048 | 0.6760519 |
| TCGA-EM-A3AL-01A | 0.469526419 | 0.414897242 | 0.631796268 | 0.502351194 | 0.438961533 | 0.7284653 |
| TCGA-EM-A3AN-01A | 0.520992117 | 0.455106453 | 0.643130806 | 0.490871666 | 0.473172221 | 0.6974318 |
| TCGA-EM-A3AO-01A | 0.556633122 | 0.547454794 | 0.65671852 | 0.480835997 | 0.494005259 | 0.6609699 |
| TCGA-EM-A3AP-01A | 0.544317993 | 0.423049589 | 0.652873924 | 0.503941723 | 0.462082725 | 0.6986617 |
| TCGA-EM-A3AQ-01A | 0.533036922 | 0.465781821 | 0.628620085 | 0.486199294 | 0.462760581 | 0.6782349 |
| TCGA-EM-A3AR-01A | 0.485943662 | 0.422854199 | 0.626746491 | 0.48517689 | 0.458139178 | 0.6996464 |
| TCGA-EM-A3FJ-01A | 0.513148238 | 0.441126006 | 0.631855937 | 0.477406144 | 0.474485035 | 0.7035372 |
| TCGA-EM-A3FK-01A | 0.540962672 | 0.427116812 | 0.661080286 | 0.486017954 | 0.464995176 | 0.7073456 |
| TCGA-EM-A3FL-01A | 0.51439491 | 0.412418464 | 0.631594605 | 0.498636754 | 0.439976428 | 0.7519988 |
| TCGA-EM-A3FM-01A | 0.538019067 | 0.477160919 | 0.647090779 | 0.475299289 | 0.503597546 | 0.6821048 |
| TCGA-EM-A3FN-01A | 0.475966209 | 0.413655039 | 0.624270015 | 0.487593474 | 0.430036298 | 0.7220558 |
| TCGA-EM-A3FO-01A | 0.513241498 | 0.463474595 | 0.643318865 | 0.488604763 | 0.466643008 | 0.6912646 |
| TCGA-EM-A3FP-01A | 0.568031905 | 0.485217848 | 0.64403004 | 0.488094626 | 0.483368072 | 0.709895 |
| TCGA-EM-A3FQ-01A | 0.571829655 | 0.515063288 | 0.668312815 | 0.478220023 | 0.539590286 | 0.6974708 |
| TCGA-EM-A3FR-01A | 0.475600857 | 0.415370701 | 0.627457956 | 0.494579449 | 0.438098353 | 0.6987424 |
| TCGA-EM-A3O3-01A | 0.566986469 | 0.558552097 | 0.637932564 | 0.492131349 | 0.538499453 | 0.6838216 |
| TCGA-EM-A3O6-01A | 0.517658494 | 0.42347587 | 0.622628429 | 0.509611994 | 0.432656116 | 0.7337588 |
| TCGA-EM-A3O7-01A | 0.51368533 | 0.441103195 | 0.636084854 | 0.483040057 | 0.46094725 | 0.6927295 |
| TCGA-EM-A3O8-01A | 0.465851744 | 0.422921193 | 0.621409186 | 0.511926355 | 0.422412529 | 0.6959055 |
| TCGA-EM-A3OA-01A | 0.4793972 | 0.424959804 | 0.631189494 | 0.506098105 | 0.425406471 | 0.7470304 |
| TCGA-EM-A3OB-01A | 0.492747074 | 0.416764584 | 0.624957312 | 0.508869208 | 0.423621947 | 0.6793034 |
| TCGA-EM-A3ST-01A | 0.512446694 | 0.48837726 | 0.628653557 | 0.493811161 | 0.436320673 | 0.6515543 |
| TCGA-EM-A3SU-01A | 0.559856866 | 0.549086868 | 0.650490105 | 0.48867536 | 0.520511491 | 0.716982 |
| TCGA-EM-A3SX-01A | 0.513416006 | 0.457007918 | 0.639412382 | 0.500142913 | 0.453336662 | 0.6780502 |
| TCGA-EM-A3SY-01A | 0.508581514 | 0.448506829 | 0.642929351 | 0.514963912 | 0.461906173 | 0.7796943 |
| TCGA-EM-A3SZ-01A | 0.56487019 | 0.494539179 | 0.668140683 | 0.494150292 | 0.510121915 | 0.716337 |
| TCGA-EM-A4FF-01A | 0.541302159 | 0.496332881 | 0.656259913 | 0.491542934 | 0.492795053 | 0.6896189 |
| TCGA-EM-A4FH-01A | 0.505991292 | 0.405617994 | 0.622199758 | 0.495180209 | 0.416351603 | 0.7194971 |
| TCGA-EM-A4FK-01A | 0.470084373 | 0.406698295 | 0.638731602 | 0.503022337 | 0.428553654 | 0.731913 |
| TCGA-EM-A4FM-01A | 0.524676284 | 0.483526204 | 0.648268612 | 0.473553631 | 0.501276485 | 0.6954086 |
| TCGA-EM-A4FN-01A | 0.533668969 | 0.476082162 | 0.642537422 | 0.475559553 | 0.492158461 | 0.6891543 |
| TCGA-EM-A4FO-01A | 0.566684775 | 0.563226265 | 0.666912474 | 0.486688541 | 0.536067866 | 0.6780418 |
| TCGA-EM-A4FQ-01A | 0.540647465 | 0.504968545 | 0.650059997 | 0.481391026 | 0.499851359 | 0.6839786 |
| TCGA-EM-A4FR-01A | 0.497184429 | 0.492367747 | 0.626258085 | 0.487464226 | 0.444203356 | 0.67347 |
| TCGA-EM-A4FU-01A | 0.513618857 | 0.461474472 | 0.630388969 | 0.507734206 | 0.454007521 | 0.6921483 |
| TCGA-EM-A4FV-01A | 0.551151386 | 0.517369544 | 0.65509293 | 0.492440335 | 0.497471359 | 0.6827437 |
| TCGA-EM-A4G1-01A | 0.519672995 | 0.454898893 | 0.643074304 | 0.500461266 | 0.472722521 | 0.7101237 |
| TCGA-ET-A25G-01A | 0.538342884 | 0.434574933 | 0.666011703 | 0.488939724 | 0.493399662 | 0.6886314 |
| TCGA-ET-A25I-01A | 0.523292928 | 0.469858488 | 0.640521653 | 0.49524085 | 0.471838243 | 0.7175241 |
| TCGA-ET-A25K-01A | 0.515261862 | 0.44122502 | 0.653874403 | 0.482816166 | 0.473982585 | 0.6573513 |
| TCGA-ET-A25L-01A | 0.538791862 | 0.469956507 | 0.655302137 | 0.479468071 | 0.493907401 | 0.6874449 |
| TCGA-ET-A25M-01A | 0.491271502 | 0.417245937 | 0.646984713 | 0.473830658 | 0.473288055 | 0.6746843 |
| TCGA-ET-A25N-01A | 0.514799001 | 0.430196063 | 0.663226784 | 0.484232057 | 0.49779775 | 0.6928969 |
| TCGA-ET-A25O-01A | 0.486408527 | 0.413098355 | 0.63009701 | 0.49431863 | 0.437384349 | 0.7044719 |
| TCGA-ET-A25P-01A | 0.492203718 | 0.408638808 | 0.637334447 | 0.506734654 | 0.443857171 | 0.6820608 |
| TCGA-ET-A25R-01A | 0.54547746 | 0.459150409 | 0.654854262 | 0.477955604 | 0.496105923 | 0.6668081 |
| TCGA-ET-A2MX-01A | 0.514884821 | 0.422590315 | 0.649285928 | 0.495674008 | 0.470826228 | 0.7449763 |
| TCGA-ET-A2MY-01A | 0.505145933 | 0.429271969 | 0.640728367 | 0.494912565 | 0.46816737 | 0.7074485 |
| TCGA-ET-A2MZ-01A | 0.521869193 | 0.434078724 | 0.644996147 | 0.485633452 | 0.460775396 | 0.6806701 |
| TCGA-ET-A2N0-01A | 0.525376321 | 0.486321499 | 0.628747691 | 0.480524981 | 0.500905971 | 0.6875524 |
| TCGA-ET-A2N3-01B | 0.504585741 | 0.417973743 | 0.615946496 | 0.490382591 | 0.42193696 | 0.6855105 |
| TCGA-ET-A2N4-01A | 0.507312081 | 0.460322191 | 0.626849926 | 0.501922882 | 0.45108131 | 0.6724769 |
| TCGA-ET-A2N5-01A | 0.485707397 | 0.430173498 | 0.629667058 | 0.510624199 | 0.443371698 | 0.7115359 |
| TCGA-ET-A39I-01A | 0.505588476 | 0.40767641 | 0.632095067 | 0.502014512 | 0.435229313 | 0.7030485 |
| TCGA-ET-A39J-01A | 0.515590184 | 0.431120234 | 0.647839672 | 0.48128992 | 0.469933041 | 0.6811743 |
| TCGA-ET-A39K-01A | 0.533448384 | 0.501514604 | 0.639736582 | 0.477947348 | 0.4936213 | 0.6780894 |
| TCGA-ET-A39L-01A | 0.490169499 | 0.427056613 | 0.636847736 | 0.48052776 | 0.458657546 | 0.6868822 |
| TCGA-ET-A39M-01A | 0.573149973 | 0.564134844 | 0.647821334 | 0.484424272 | 0.530664664 | 0.6677488 |
| TCGA-ET-A39N-01A | 0.525795525 | 0.470708933 | 0.637411849 | 0.486213371 | 0.462426065 | 0.6744931 |
| TCGA-ET-A39O-01A | 0.574127519 | 0.502633995 | 0.663741051 | 0.494504587 | 0.511284601 | 0.6904591 |
| TCGA-ET-A39P-01A | 0.516308123 | 0.431183217 | 0.640694974 | 0.487326025 | 0.46758377 | 0.6970128 |
| TCGA-ET-A39R-01A | 0.584912554 | 0.540212834 | 0.666266591 | 0.498437104 | 0.526681201 | 0.6728007 |
| TCGA-ET-A39S-01A | 0.506605177 | 0.430521179 | 0.641326984 | 0.490287177 | 0.478630052 | 0.6941387 |
| TCGA-ET-A39T-01A | 0.504257902 | 0.429875859 | 0.642574949 | 0.48808676 | 0.463581423 | 0.707729 |
| TCGA-ET-A3BN-01A | 0.519448784 | 0.415314029 | 0.663120612 | 0.494693104 | 0.478643434 | 0.6945377 |
| TCGA-ET-A3BO-01A | 0.549886887 | 0.50648324 | 0.662519167 | 0.489243473 | 0.51098948 | 0.671267 |
| TCGA-ET-A3BP-01A | 0.509637034 | 0.451197882 | 0.640067379 | 0.486127528 | 0.465021774 | 0.6894824 |
| TCGA-ET-A3BQ-01B | 0.516501082 | 0.458948242 | 0.648930821 | 0.483915055 | 0.48076436 | 0.6676117 |
| TCGA-ET-A3BS-01A | 0.547738361 | 0.541524748 | 0.660800796 | 0.484941573 | 0.521848244 | 0.6990372 |
| TCGA-ET-A3BT-01A | 0.516638163 | 0.453300151 | 0.64268051 | 0.485537903 | 0.486614749 | 0.686943 |
| TCGA-ET-A3BU-01A | 0.535007489 | 0.460780712 | 0.662429434 | 0.485838392 | 0.489418617 | 0.6681284 |
| TCGA-ET-A3BV-01A | 0.553571713 | 0.452976653 | 0.676016249 | 0.472446186 | 0.533284995 | 0.6595973 |
| TCGA-ET-A3BW-01A | 0.4883869 | 0.434760094 | 0.628198533 | 0.487583267 | 0.442538508 | 0.6728237 |
| TCGA-ET-A3BX-01A | 0.534493288 | 0.456929551 | 0.672512784 | 0.498769128 | 0.497405972 | 0.6963244 |
| TCGA-ET-A3DO-01A | 0.59109043 | 0.531541406 | 0.681102933 | 0.492583577 | 0.552721939 | 0.6914871 |
| TCGA-ET-A3DP-01A | 0.498658021 | 0.452435896 | 0.654282751 | 0.476326942 | 0.486769698 | 0.6509868 |
| TCGA-ET-A3DQ-01A | 0.496648267 | 0.457607626 | 0.663963763 | 0.482216594 | 0.511347151 | 0.6826764 |
| TCGA-ET-A3DR-01A | 0.473201028 | 0.410366068 | 0.646080442 | 0.489265796 | 0.458120846 | 0.6974805 |
| TCGA-ET-A3DS-01A | 0.496694682 | 0.425149683 | 0.627683034 | 0.51952174 | 0.455255748 | 0.6988574 |
| TCGA-ET-A3DU-01A | 0.607478405 | 0.569168 | 0.683881725 | 0.476523097 | 0.561077716 | 0.6801725 |
| TCGA-ET-A3DV-01A | 0.500822123 | 0.411789309 | 0.624720214 | 0.499068941 | 0.434895414 | 0.7008428 |
| TCGA-ET-A3DW-01A | 0.558175484 | 0.475430173 | 0.653851282 | 0.49015524 | 0.492624498 | 0.7218796 |
| TCGA-ET-A40P-01A | 0.520464552 | 0.506147445 | 0.629052574 | 0.490713856 | 0.465686432 | 0.6858633 |
| TCGA-ET-A40Q-01A | 0.579877893 | 0.494866094 | 0.655572692 | 0.493381682 | 0.508749424 | 0.6932479 |
| TCGA-ET-A40R-01A | 0.503049455 | 0.489704822 | 0.645746548 | 0.493295483 | 0.490392554 | 0.6842918 |
| TCGA-ET-A40S-01A | 0.594706903 | 0.511957417 | 0.658226027 | 0.490907474 | 0.520075627 | 0.6621716 |
| TCGA-ET-A40T-01A | 0.554383072 | 0.510793342 | 0.653146257 | 0.489837233 | 0.499040956 | 0.6614475 |
| TCGA-ET-A4KN-01A | 0.500918581 | 0.548303528 | 0.626463052 | 0.45407727 | 0.532853322 | 0.6828524 |
| TCGA-ET-A4KQ-01A | 0.505041396 | 0.406710716 | 0.632011834 | 0.498184715 | 0.434597385 | 0.6833059 |
| TCGA-FE-A22Z-01A | 0.49726581 | 0.4564619 | 0.653240694 | 0.47163374 | 0.480280933 | 0.6890286 |
| TCGA-FE-A230-01A | 0.554988837 | 0.491524772 | 0.666098996 | 0.485768637 | 0.516202456 | 0.6671434 |
| TCGA-FE-A231-01A | 0.557701275 | 0.447294351 | 0.636243878 | 0.489341152 | 0.480820244 | 0.7040378 |
| TCGA-FE-A232-01A | 0.539433657 | 0.475812082 | 0.644898944 | 0.486679605 | 0.500406748 | 0.6939754 |
| TCGA-FE-A233-01A | 0.52713539 | 0.421874992 | 0.659425167 | 0.479439447 | 0.47487865 | 0.6766262 |
| TCGA-FE-A234-01A | 0.544731182 | 0.452768981 | 0.662845561 | 0.491019842 | 0.504459527 | 0.6891967 |
| TCGA-FE-A235-01A | 0.503741062 | 0.470989608 | 0.640670837 | 0.476861193 | 0.501622813 | 0.6551418 |
| TCGA-FE-A236-01A | 0.520241278 | 0.407574181 | 0.649743161 | 0.480272092 | 0.459745468 | 0.6832697 |
| TCGA-FE-A237-01A | 0.519211905 | 0.442993588 | 0.650229279 | 0.482342789 | 0.48938024 | 0.679626 |
| TCGA-FE-A238-01A | 0.542023756 | 0.465166282 | 0.651610009 | 0.484522821 | 0.49147089 | 0.6862637 |
| TCGA-FE-A239-01A | 0.440155556 | 0.395446388 | 0.597977496 | 0.501636949 | 0.420885594 | 0.6469707 |
| TCGA-FE-A23A-01A | 0.577856223 | 0.518024322 | 0.67381614 | 0.480863476 | 0.529408811 | 0.6745706 |
| TCGA-FE-A3PA-01A | 0.544601126 | 0.49609098 | 0.632701053 | 0.474922669 | 0.465688748 | 0.657581 |
| TCGA-FE-A3PB-01A | 0.57778318 | 0.494830504 | 0.657606794 | 0.4822854 | 0.524298271 | 0.6969708 |
| TCGA-FE-A3PC-01A | 0.514736249 | 0.512638312 | 0.634697066 | 0.476932027 | 0.480940756 | 0.7182472 |
| TCGA-FE-A3PD-01A | 0.556267446 | 0.517571029 | 0.646289121 | 0.496855584 | 0.485003932 | 0.6927977 |
| TCGA-FK-A3S3-01A | 0.500440944 | 0.438354612 | 0.634281598 | 0.493422207 | 0.452055376 | 0.6620334 |
| TCGA-FK-A3SB-01A | 0.502806239 | 0.440620912 | 0.64505825 | 0.480766555 | 0.47347904 | 0.6594345 |
| TCGA-FK-A3SD-01A | 0.50888298 | 0.400111452 | 0.616841021 | 0.503086577 | 0.417566904 | 0.7073041 |
| TCGA-FK-A3SE-01A | 0.532410895 | 0.440486614 | 0.6573248 | 0.50248636 | 0.488254318 | 0.7022369 |
| TCGA-FK-A3SG-01A | 0.529087326 | 0.443102449 | 0.642282126 | 0.487652582 | 0.481183154 | 0.692695 |
| TCGA-FK-A3SH-01A | 0.493555721 | 0.471642365 | 0.633686326 | 0.477140753 | 0.455279546 | 0.6464224 |
| TCGA-FK-A4UB-01A | 0.473316328 | 0.46980741 | 0.622458016 | 0.468915734 | 0.456449395 | 0.6650547 |
| TCGA-FY-A2QD-01A | 0.511423885 | 0.382981454 | 0.627926384 | 0.503587474 | 0.422895792 | 0.725915 |
| TCGA-FY-A3BL-01A | 0.511542044 | 0.423425998 | 0.641429455 | 0.491735147 | 0.457911461 | 0.7057045 |
| TCGA-FY-A3I4-01A | 0.547313946 | 0.48413653 | 0.656397204 | 0.47181338 | 0.502801124 | 0.643561 |
| TCGA-FY-A3I5-01B | 0.477856358 | 0.449048509 | 0.629885363 | 0.509004634 | 0.482544455 | 0.6989036 |
| TCGA-FY-A3NM-01A | 0.487341785 | 0.426622438 | 0.631856691 | 0.509117998 | 0.423967787 | 0.7616947 |
| TCGA-FY-A3NP-01A | 0.520328778 | 0.41277162 | 0.637044117 | 0.501317795 | 0.431426281 | 0.6925729 |
| TCGA-FY-A3ON-01A | 0.55919306 | 0.502620365 | 0.662147683 | 0.493301319 | 0.497525001 | 0.6880957 |
| TCGA-FY-A3R6-01A | 0.598404909 | 0.526463515 | 0.673941146 | 0.484991657 | 0.529743519 | 0.6842793 |
| TCGA-FY-A3R7-01A | 0.546452508 | 0.45384654 | 0.653548138 | 0.486515076 | 0.48375995 | 0.6904856 |
| TCGA-FY-A3R8-01A | 0.549224141 | 0.482761937 | 0.65284802 | 0.493889715 | 0.498640369 | 0.6948304 |
| TCGA-FY-A3R9-01A | 0.489439936 | 0.40123256 | 0.627361341 | 0.517462209 | 0.434712223 | 0.7579204 |
| TCGA-FY-A3RA-01A | 0.512835265 | 0.423691863 | 0.649948628 | 0.49001389 | 0.468851482 | 0.7192864 |
| TCGA-FY-A3TY-01A | 0.541074496 | 0.488913947 | 0.663114939 | 0.481150331 | 0.525806006 | 0.6853748 |
| TCGA-FY-A3W9-01A | 0.477041493 | 0.439678389 | 0.634086044 | 0.499907799 | 0.440140081 | 0.6794547 |
| TCGA-FY-A3WA-01A | 0.493634575 | 0.439351576 | 0.629993491 | 0.504963032 | 0.438888986 | 0.6949392 |
| TCGA-FY-A3YR-01A | 0.556222851 | 0.459500532 | 0.65637098 | 0.489664633 | 0.497353273 | 0.7161852 |
| TCGA-FY-A40K-01A | 0.531080911 | 0.491491467 | 0.64796492 | 0.479215026 | 0.494232499 | 0.6751897 |
| TCGA-FY-A40L-01A | 0.558250592 | 0.475710049 | 0.652423381 | 0.496040528 | 0.497085441 | 0.693899 |
| TCGA-FY-A40M-01A | 0.535263401 | 0.454489908 | 0.635979177 | 0.481271052 | 0.439104273 | 0.6393718 |
| TCGA-FY-A40N-01A | 0.547200771 | 0.481557056 | 0.642341669 | 0.498394734 | 0.464111051 | 0.6980713 |
| TCGA-FY-A4B0-01A | 0.489099091 | 0.493944022 | 0.628877724 | 0.497197775 | 0.463951548 | 0.7299184 |
| TCGA-FY-A4B3-01A | 0.534936971 | 0.449942466 | 0.658900282 | 0.487298154 | 0.501778178 | 0.6445224 |
| TCGA-FY-A4B4-01A | 0.577759563 | 0.497631044 | 0.662782414 | 0.490377766 | 0.517635484 | 0.6748984 |
| TCGA-FY-A76V-01A | 0.509351555 | 0.437823353 | 0.645609351 | 0.489870209 | 0.469663514 | 0.678019 |
| TCGA-GE-A2C6-01A | 0.533786246 | 0.506632475 | 0.656368951 | 0.496025471 | 0.501423278 | 0.6584582 |
| TCGA-H2-A26U-01A | 0.602207795 | 0.532196875 | 0.679391988 | 0.47919699 | 0.548438067 | 0.6686002 |
| TCGA-H2-A2K9-01A | 0.498741581 | 0.388755386 | 0.640341052 | 0.505748207 | 0.430942171 | 0.6850456 |
| TCGA-H2-A3RH-01A | 0.543996181 | 0.457420425 | 0.642957896 | 0.499126852 | 0.449775509 | 0.6706027 |
| TCGA-H2-A3RI-01A | 0.510951182 | 0.463597919 | 0.635653417 | 0.49253411 | 0.478279221 | 0.7101246 |
| TCGA-H2-A421-01A | 0.580160569 | 0.534795512 | 0.657385328 | 0.488964094 | 0.523711369 | 0.6746625 |
| TCGA-H2-A422-01A | 0.526043835 | 0.437569088 | 0.654594754 | 0.496287104 | 0.468560292 | 0.7059227 |
| TCGA-IM-A3EB-01A | 0.587044151 | 0.503353868 | 0.666639597 | 0.494486922 | 0.52346906 | 0.700067 |
| TCGA-IM-A3ED-01A | 0.555476538 | 0.508144921 | 0.673331587 | 0.477024796 | 0.516735263 | 0.6831673 |
| TCGA-IM-A3U2-01A | 0.53809366 | 0.446279538 | 0.641571263 | 0.482314018 | 0.492134231 | 0.7120918 |
| TCGA-IM-A3U3-01A | 0.530296584 | 0.471433669 | 0.655504677 | 0.486609353 | 0.487999512 | 0.6785801 |
| TCGA-IM-A41Z-01A | 0.553306392 | 0.486868003 | 0.644864751 | 0.511050669 | 0.478674386 | 0.7169903 |
| TCGA-IM-A420-01A | 0.534569142 | 0.521703109 | 0.666441179 | 0.488241137 | 0.52266742 | 0.69074 |
| TCGA-J8-A3NZ-01A | 0.5418161 | 0.458507929 | 0.665128167 | 0.484389577 | 0.497600396 | 0.6943406 |
| TCGA-J8-A3O0-01A | 0.536054079 | 0.446753162 | 0.652491944 | 0.505975068 | 0.469074149 | 0.6789254 |
| TCGA-J8-A3O1-01A | 0.518197917 | 0.423154559 | 0.661337167 | 0.490767752 | 0.462502186 | 0.7116398 |
| TCGA-J8-A3O2-01A | 0.529993293 | 0.463189722 | 0.654496608 | 0.486017206 | 0.500427101 | 0.6820516 |
| TCGA-J8-A3YD-01A | 0.511184692 | 0.424365308 | 0.642940099 | 0.499696791 | 0.447959976 | 0.7204689 |
| TCGA-J8-A3YE-01A | 0.508105461 | 0.420999032 | 0.641019495 | 0.498564475 | 0.441302985 | 0.7304249 |
| TCGA-J8-A3YF-01A | 0.572415483 | 0.515241834 | 0.654926109 | 0.490634023 | 0.511102239 | 0.6885462 |
| TCGA-J8-A3YG-01A | 0.561481626 | 0.474434387 | 0.656445902 | 0.486668713 | 0.500879638 | 0.673653 |
| TCGA-J8-A3YH-01A | 0.528911662 | 0.470753646 | 0.634017601 | 0.489939825 | 0.459675679 | 0.6665333 |
| TCGA-J8-A42S-01A | 0.506867006 | 0.436526954 | 0.638305536 | 0.481115089 | 0.478028699 | 0.6713232 |
| TCGA-J8-A4HW-01A | 0.505867164 | 0.417034179 | 0.64982679 | 0.495508877 | 0.455866967 | 0.70732 |
| TCGA-J8-A4HY-01A | 0.51004904 | 0.451167165 | 0.636116209 | 0.489209789 | 0.468448785 | 0.7206718 |
| TCGA-KS-A41F-01A | 0.466937212 | 0.443080361 | 0.616101259 | 0.466168378 | 0.445853609 | 0.6635847 |
| TCGA-KS-A41I-01A | 0.510122303 | 0.511793234 | 0.62750798 | 0.475608002 | 0.480001482 | 0.6473709 |
| TCGA-KS-A41J-01A | 0.550914671 | 0.509825117 | 0.646526107 | 0.487344675 | 0.505899934 | 0.6926174 |
| TCGA-KS-A41L-01A | 0.524630311 | 0.434931139 | 0.62901501 | 0.491786926 | 0.430097757 | 0.716501 |
| TCGA-KS-A4I1-01A | 0.499622765 | 0.498980227 | 0.635546202 | 0.463540508 | 0.466516637 | 0.6566456 |
| TCGA-KS-A4I3-01A | 0.523365929 | 0.430089919 | 0.649515093 | 0.485870415 | 0.470008333 | 0.7046788 |
| TCGA-KS-A4I5-01A | 0.550067741 | 0.47177441 | 0.650497798 | 0.472935356 | 0.490015611 | 0.6664258 |
| TCGA-KS-A4I7-01A | 0.555282417 | 0.465905832 | 0.637440991 | 0.489094008 | 0.488989898 | 0.6771278 |
| TCGA-KS-A4I9-01A | 0.547956433 | 0.455285641 | 0.65342306 | 0.48885347 | 0.489453074 | 0.7248653 |
| TCGA-KS-A4IB-01A | 0.533200967 | 0.444207397 | 0.638873381 | 0.501750437 | 0.449311951 | 0.7036826 |
| TCGA-KS-A4IC-01A | 0.522851674 | 0.46698322 | 0.642174851 | 0.479723827 | 0.479230275 | 0.6934807 |
| TCGA-KS-A4ID-01A | 0.575973309 | 0.5462667 | 0.65779566 | 0.487820257 | 0.522264616 | 0.6980731 |
| TCGA-L6-A4EP-01A | 0.521035545 | 0.494828156 | 0.629615179 | 0.472962793 | 0.482108807 | 0.6660613 |
| TCGA-L6-A4EQ-01A | 0.494338617 | 0.463995927 | 0.628674006 | 0.485117327 | 0.467030333 | 0.6677291 |
| TCGA-L6-A4ET-01A | 0.499955543 | 0.435192203 | 0.628720813 | 0.480580638 | 0.450087446 | 0.6716527 |
| TCGA-L6-A4EU-01A | 0.535683128 | 0.482625428 | 0.648269868 | 0.487297687 | 0.516054955 | 0.7017819 |
| TCGA-MK-A4N6-01A | 0.510188496 | 0.486281617 | 0.666203097 | 0.489937003 | 0.512539357 | 0.6638378 |
| TCGA-MK-A4N7-01A | 0.47049367 | 0.439022782 | 0.640756116 | 0.491908035 | 0.463475164 | 0.6751819 |
| TCGA-MK-A4N9-01A | 0.487372605 | 0.452704388 | 0.630794462 | 0.48582502 | 0.460506236 | 0.6806782 |
| TCGA-MK-A84Z-01A | 0.491479038 | 0.443903578 | 0.631411468 | 0.500629187 | 0.436571069 | 0.6766134 |
| TCGA-QD-A8IV-01A | 0.489126385 | 0.473244427 | 0.636186002 | 0.50472553 | 0.44942397 | 0.6863516 |
